# *microRNA-206* modulates an Rtn4a/Cxcr4a/Thbs3a axis in newly forming somites to maintain and stabilize the somite boundary formation of zebrafish embryos

**DOI:** 10.1098/rsob.170009

**Published:** 2017-07-12

**Authors:** Cheng-Yung Lin, Jun-Yu He, Chih-Wei Zeng, Moo-Rumg Loo, Wen-Yen Chang, Po-Hsiang Zhang, Huai-Jen Tsai

**Affiliations:** 1 Institute of Biomedical Sciences, Mackay Medical College, No. 46, Section 3 Zhongzhen Road, Sanzhi Dist., New Taipei City 252, Taiwan, Republic of China; 2 Institute of Molecular and Cellular Biology, National Taiwan University, No. 1, Section 4, Roosevelt Road, Taipei 106, Taiwan, Republic of China

**Keywords:** zebrafish, somite boundary formation, *miR-206*, *rtn4a*, *cxcr4a*, *thbs3a*

## Abstract

Although *microRNA-206* (*miR-206*) is known to regulate proliferation and differentiation of muscle fibroblasts, the role of *miR-206* in early-stage somite development is still unknown. During somitogenesis of zebrafish embryos, *reticulon4a* (*rtn4a*) is specifically repressed by *miR-206.* The somite boundary was defective, and actin filaments were crossing over the boundary in either *miR-206*-knockdown or *rtn4a*-overexpressed embryos. In these treated embryos, C–X–C motif chemokine receptor 4a (*cxcr4a*) was reduced, while thrombospondin 3a (*thbs3a*) was increased. The defective boundary was phenocopied in either *cxcr4a*-knockdown or *thbs3a-*overexpressed embryos. Repression of *thbs3a* expression by *cxcr4a* reduced the occurrence of the boundary defect. We demonstrated that *cxcr4a* is an upstream regulator of *thbs3a* and that defective boundary cells could not process epithelialization in the absence of intracellular accumulation of the phosphorylated focal adhesion kinase (p-FAK) in boundary cells. Therefore, in the newly forming somites, *miR-206-*mediated downregulation of *rtn4a* increases *cxcr4a.* This activity largely decreases *thbs3a* expression in the epithelial cells of the somite boundary, which causes epithelialization of boundary cells through mesenchymal–epithelial transition (MET) and eventually leads to somite boundary formation. Collectively, we suggest that *miR-206* mediates a novel pathway, the Rtn4a/Cxcr4a/Thbs3a axis, that allows boundary cells to undergo MET and form somite boundaries in the newly forming somites of zebrafish embryos.

## 1. Introduction

MicroRNAs (miRNAs) are short (approx. 22 nt) endogenous non-coding RNAs that regulate gene expression at the post-transcriptional level by silencing target gene(s) through pairing between the seed sequence(s) of miRNA and the 3′-untranslated region (3′UTR) of target messenger RNAs (mRNAs). To promote dynamic equilibrium of expression among genes, miRNAs play an important role in cell differentiation, tissue identity [1] and normal development [2]. In particular, *microRNA-206* (*miR-206*) has been reported as a regulator of muscle proliferation and differentiation, but its function in the mesoderm and somite cells of embryos remains unclear. Importantly, *miR-206* can be detected at the one-cell stage of zebrafish embryos [3], and its expression increases in somites between 12 and 16 hpf [4,5]. Therefore, we employed the labelled microRNA pull-down (LAMP) assay [6] of mRNAs extracted from 16-hpf zebrafish embryos and found that *reticulon 4a* (*rtn4a*) is a target gene for *miR-206* at this developmental stage. Zebrafish Rtn4a is essential for embryonic development and patterning of the nervous system [7,8]. However, the role Rtn4a plays at the early stage of somite boundary formation has not been elucidated.

Somite boundary formation of vertebrates is an example of developmental mesenchymal–epithelial transition (MET). More specifically, the presomitic mesoderm (PSM), an area of mesoderm in the neurulating embryo, consists primarily of mesenchymal cells. These mesenchymal cells surrounding the PSM become epithelial cells through MET and separate from the PSM to form somites [9–11]. Somites are transient structures that are present on both sides of the body axis from head to tail, first forming a repetitive and metameric configuration and later differentiating into skin, skeletal muscle and axial bone in late embryogenesis. As somites separate from each other, a morphological boundary is formed, termed the gap or cleft [9–11]. Therefore, we can define somite formation as the reiterated subdivision of paraxial mesoderm into paired, epithelial spheres of cells on either side of the midline [12]. Studies reveal a pre-patterning process in the anterior of the PSM before the morphological appearance of somite pairs. Cooke & Zeeman [13] proposed a clock and wavefront model to explain the pattern formation of PSM. They explain that a clock mechanism controls cell oscillations between anterior and posterior somitic identities in the PSM. During this process, the position of future somite boundaries is selected in the PSM. Both anterior and posterior somitic identities are responsible for boundary formation. Therefore, this boundary formation process makes the vertebrate a particularly good model with which to study MET [14].

Importantly, the boundary formation process may be considered the product of a two-step signalling cascade. The first step ensures normal development of somite and new boundary formation, and the second ensures proper maintenance of the boundary gap. In zebrafish, during the first developmental process, fluctuate expression patterns of two Hes-related genes, *her1* and *her7*, oscillate in PSM, known as the segmentation clock genes, controlling and coordinating the orderly process of oscillation [15,16]. Expression of Notch ligand DeltaC also oscillates during somitogenesis [17,18]. The oscillation phase of DeltaC expression is synchronized with that of *her1* and *her7*. The *tbx6* gene is an essential factor for the formation of the somite boundary [19], because the Tbx6 protein domain defines the position of the succeeding somite boundary that will be formed during orderly somite segmentation. Furthermore, *ripply1* and *ripply2* restrict *tbx6* expression in the anterior edge of newly forming somites [20]. Particularly, in zebrafish, Mesp is not essential for Ripply-dependent boundary positioning, while it is required for the generation of morphological boundary and rostro-caudal polarity formation [21].

To maintain the boundary gap in the newly formed boundary, somite cells produce extracellular matrix (ECM) to form muscle plasticity and myotendinous junction (MTJ) [22]. Unlike amniotes, such as mouse and chicken, zebrafish undergoes simultaneous epithelialization at both anterior and posterior border cells [11]. Epha4 and Ephrinb2 signalling induces the MET of somite boundary formation and ECM assembly in zebrafish [23,24]. Rap1b, a GTPase, acts downstream of Ephrin reverse signalling and contributes to Integrin inside-out activation, resulting in fibronectin polymerization at somite boundaries [25]. Recently, Julich *et al*. [26] reported that Cadherin 2 (Cdh2) is also essential for the epithelialization of cells along the somite boundary. Cdh2 causes Integrin α5 inactivation within the paraxial mesoderm mesenchyme through cell–cell cohesion. When embryos start to form a new boundary, Cdh2 expression decreases along the nascent boundary, resulting in the accumulation of fibronectin. Thereafter, outside-in Integrin signalling begins to activate phosphorylated focal adhesion kinase (p-FAK) in boundary cells through the Integrin receptor [26].

Based on this foundation, we provide further insight into the molecular regulatory pathway that underlies the involvement of *miR-206* in the somite boundary formation of zebrafish embryos. Specifically, we confirm that *miR-206* plays a role in somite boundary formation at the early stage through silencing *rtn4a* expression. Furthermore, we found that C–X–C motif chemokine receptor 4a (Cxcr4a) represses the expression level of Rtn4a. Cxcr4a has been reported to be involved in somite rotation in zebrafish embryos [27] and somite morphogenesis in *Xenopus laevis* embryos [28]. Knockdown of *cxcr4a* in *Xenopus* resulted in defective formation of the somite boundary [28]. Additionally, we demonstrated that Cxcr4a is able to repress the expression of thrombospondin 3a (Thbs3a), an ECM protein. Finally, we proved that Thbs3a is involved in mediating the epithelialization of somite boundary cells that affect somite boundary formation. Thus, for the first time, we have demonstrated that a *miR-206/rtn4a/cxcr4a/thbs3a* axis is also importantly involved in controlling somite boundary formation during somitogenesis of zebrafish embryos.

## 2. Material and methods

### 2.1. Zebrafish husbandry and microscopy observation

Wild-type zebrafish (*Danio rerio*) AB strain (University of Oregon) and transgenic lines *Tg(myf5:GFP)* [29] and *Tg*(*α-actin:RFP*) [30] were used. Production and stage identification of embryos followed the description by Westerfield [31] and Kimmel *et al.* [32]. Microscopy observation was performed with a fluorescent stereomicroscope (Leica) and a confocal spectral microscope (Nikon).

### 2.2. Searching for the putative target genes of *miR-206*

To search for the putative target genes of *miR-206*, we performed LAMP assay [6] with some modifications. The pre-*miR-206* was labelled with biotin and then mixed with cell extracts. The putative target genes were precipitated by anti-biotin agarose beads (Sigma) and transformed into cDNA by reverse transcriptase-polymerase chain reaction (RT-PCR). Finally, these putative cDNAs for *miR-206-*targeting were further combined with Zebrafish Whole Genome Microarray (Agilent).

### 2.3. Plasmid constructs

We designed primers to perform PCR from the cDNA library of zebrafish embryos at 20 hpf to clone the complete 3′UTR segment of each cDNA of *cited3* (NM200078, +942 to +1638), *gadd45ab* (NM001002216, +587 to +1217), *znf142* (XM684944, +4436 to +5557) and *rtn4a* (NM001079912, +717 to +2082). Each PCR product was ligated into the downstream of luciferase (*luc*) gene in plasmid phRG-TK and designated as plasmid phRG-TK-*cited3*-3′UTR, -*gadd45ab*-3′UTR, -*znf142*-3′UTR and -*rtn4a*-3′UTR, respectively. The 3′UTR sequence of each gene was driven by thymidine kinase (TK) promoter. Plasmids phRL-Myf5-*cited3*-3′UTR, phRL-Myf5-*gadd45ab*-3′UTR, phRL-*znf142*-3′UTR and phRL-Myf5-*rtn4a*-3′UTR containing *cited3-*, *gadd45ab*-, *znf142*- and *rtn4a*-3′UTR sequences, respectively, were driven by the upstream regulatory elements of zebrafish Myogenic Factor 5 (Myf5) gene [33].

### 2.4. Validation of *miR-206*-targeting genes by *luc* activity assay

Dual *luc* reporter assay (Promega) was carried out in cell lines HEK-293T and C2C12 and zebrafish embryos by following the method described previously [5] with some modifications. We co-transfected 40 ng of plasmid pGL3-TK, which served as an internal control, 200 ng of each examined plasmid, including phRG-TK, phRG-TK-*cited3*-3′UTR, phRG-TK-*gadd45ab*-3′UTR, phRG-TK-*znf142*-3′UTR and phRG-TK-*rtn4a*-3′UTR, and 2 µg of plasmid pCS2-*miR-206.* The *luc* activity obtained from phRG-TK alone was the control group, which was normalized as 100%. In zebrafish embryos, we co-injected 5 ng µl^−1^ of pGL3-TK, which also served as an internal control, 5 ng µl^−1^ of each examined plasmid, including phRL-Myf5, phRL-Myf5-*cited3*-3′UTR, phRL-Myf5-*gadd45ab*-3′UTR, phRL-*znf142*-3′UTR and phRL-Myf5-*rtn4a*-3′UTR, and 200 pg of synthesized pre-*miR-206* or pre-*miR-1* RNA. The *luc* assay was performed at 20 h post-injection for 60 embryos which were randomly collected from 100 to 150 injected embryos and divided into three groups (20 embryos per group). The *luc* activity obtained from injection of phRL-Myf5 was the control group, which was normalized as 100%. The change of *luc* activity was calculated as follows: fold change = [(*Renilla* *luc* + *miR*)/(firefly *luc* + *miR*)]. Data of each group were represented as the average of three independent experiments.

### 2.5. Antisense morpholino oligonucleotides used to perform knockdown experiments

All morpholino oligonucleotides (MOs) were purchased from Gene Tools (USA) and prepared according to the protocol published by Gene Tools. The sequence and injected amount of each MO were as follows: *miR-1*-MO (AATACATACTTCTTTACATTCCA, 8 ng) [5], *miR206*-MO (GATCTCACTGAAGCCACACACTTCC, 8 ng) [5], *miR206*-*5-mis*-MO (GATATCAATGAACCCAAACAATTCC, 8 ng) (the mismatched nucleotides are underlined) [5], *rtn4a*-MO (GAAAACAAACAAACCTTGAGCGAGT, 2 ng), *cxcr4a*-MO (AGAAGTCTTTTAGAGATGGCTTAT, 8 ng) [34], and *thbs3a*-MO (AGTAAAAGGCGAAAGATTTGTGCGT, 1 ng).

### 2.6. RNA preparation and mRNA overexpression

RNA and capped mRNAs were synthesized according to the manufacturer's protocol (Epicentre). The resultant RNAs were diluted with distilled water for final molecular mass of microinjection into one embryo as follows: pre-*miR-206* RNA, 200 pg; pre-*miR-1* RNA, 200 pg; *cited3* mRNA, 200 pg; *rtn4al* mRNA, 200 pg; *rtn4am* mRNA, 200 pg; *rtn4an* mRNA, 200 pg; and *thbs3a* mRNA, 400 pg.

### 2.7. Fluorescence-activated cell sorting

The dissociation procedure of zebrafish embryonic cells was modified from Lee *et al*. [35]. Briefly, the *miR-206*-MO-injected and *rtn4al-*mRNA-injected embryos from *Tg*(*myf5:GFP*) at 20 hpf were incubated with trypsin (Sigma; 59427C) for 20 min at room temperature. Embryos were shattered by pipetting to completely separate cells from the tissue. Then, the GFP(+) cells were sorted by a cell sorter (BD FACSAria III). The GFP(+) cells were collected in TRIzol solution (Thermo Fisher Scientific) for RNA extraction.

### 2.8. Whole-mount *in situ* hybridization

Whole-mount *in situ* hybridization (WISH) followed the method described previously by Lin *et al.* [30] with exceptions. The 22-nt antisense sequences of *miR-206* (EXIQON) [5] and the cDNA coding for *rtn4al* (NM001079912), *cxcr4a* (NM131882), *thbs3a* (NM173225), *fgf8* (NM131281), *deltad* (NM130955), *her1* (NM131078), *tbx6* (NM153666), *mespa* (NM131551), *mespb* (NM131552), *dgcr8* (NM001122749), *pomt1* (NM001048067), *nkiras2* (NM001003433), *zgc56251* (BC046025), *sall4* (NM001080609) or *sdc4* (NM001048149) were used as probes.

### 2.9. Immunohistochemistry

Immunohistochemistry was performed according to the protocol described previously by Koshida *et al.* [36] with some modifications. In this study, antibodies such as anti-fibronectin (Sigma; 1 : 200), anti-γ-tubulin (Sigma; 1 : 1000), anti-laminin (Sigma; 1 : 100) and anti-phosphor FAK [pY397] (Thermo; 1 : 200), were used. Alexa 488 goat anti-rabbit IgG (Rockland) and Alexa 488 goat anti-mouse IgG (Life Technologies) served as secondary antibodies at a 1 : 1000 dilution in blocking solution. Rhodamine–phalloidin (Thermo; 1 : 200) was added in the blocking solution to detect F-actin.

### 2.10. Quantitative RT-PCR

For each experiment, we collected 100 embryos in 500 µl of Trizol reagent (Invitrogen) and stored them at −80°C. Total RNA was isolated according to the manufacturer's instructions. For quantitative RT-PCR (qPCR), first-strand cDNA was generated using 1 mg of total RNA. Both cDNA concentrations were adjusted to 200 ng ml^−1^, and q-PCR was performed using the 7900HT Fast Real-Time PCR System (Applied Biosystems, USA) according to the manufacturer's instructions. Forward and reverse primers designed for cloning each gene by PCR were as follows: GCATCAGGCACAAATTGACC and TTGAATTGCTTGTTCACCAGTC for *rtn4a*, CTGCTGGTTGCCGTATTGC and GGAATCACCTCCAGCATCA for *cxcr4a*, GAGAACATCATTTGGTCCAATC and ACCTGCTTACGGTGTGAACTG for *thbs3a*, and CTCCTCTTGGTCGCTTTGCT and CCGATTTTCTTCTCAACGCTCT for *ef1a*. Expression levels of transcripts were determined by comparison with a standard curve from total RNA isolated from wild-type (WT) embryos.

### 2.11. Western blot analysis

Total proteins extracted from embryos were analysed on a 10% SDS-PAGE followed by western blot analysis according to the procedures described by Lin *et al.* [5], except that the yolk was removed and the antibodies against Rtn4a (Abk; 1 : 1000), FAK (Cell Signaling; 1 : 1000), phosphor FAK [pY397] (Thermo; 1 : 1000), cell division control protein 42 homologue (cdc42) (New East; 1 : 500), active cdc42 (New East), α-tubulin (Sigma-Aldrich; 1 : 5000), GADPH (Santa Cruz; 1 : 1000), mouse-HRP (Santa Cruz; 1 : 5000) and rabbit-HRP (Santa Cruz; 1 : 5000) were used.

### 2.12. Defective formation of somite boundary

When embryos were injected with *miR-206*-MO, *rtn4al* mRNA, *cxcr4a*-MO and *thbs3a* mRNA, the somite boundary formation from the sixth to 20th somite of the embryos was examined at 20 and 48 hpf. We calculated the number of embryos exhibiting defective somite boundary formation, as indicated by at least one incompletely formed boundary at either side of the trunk.

## 3. Results

### 3.1. Screening of the target genes for *miR-206*

Although *miR-206* could be detected in the one-cell stage of zebrafish embryos [3], it was significantly increased in somites during developmental stages between 12 and 16 hpf [4,5]. To understand the functions of *miR-206* at that particular stage, we searched for the target genes of *miR-206* at 16 hpf through LAMP assay. In total, 117 putative target genes for *miR-206* were screened (electronic supplementary material, table S1). Four of them, including *cbp/p300-interacting transactivator with Glu/Asp-rich carboxy-terminal domain 3* (*cited3*), *growth arrest and DNA-damage-inducible alpha b* (*gadd45ab*), *zinc finger protein 142* (*znf142*) and *reticulon 4a* (*rtn4a*), were selected for further study because they were specifically expressed in somites.

**Figure 1.**
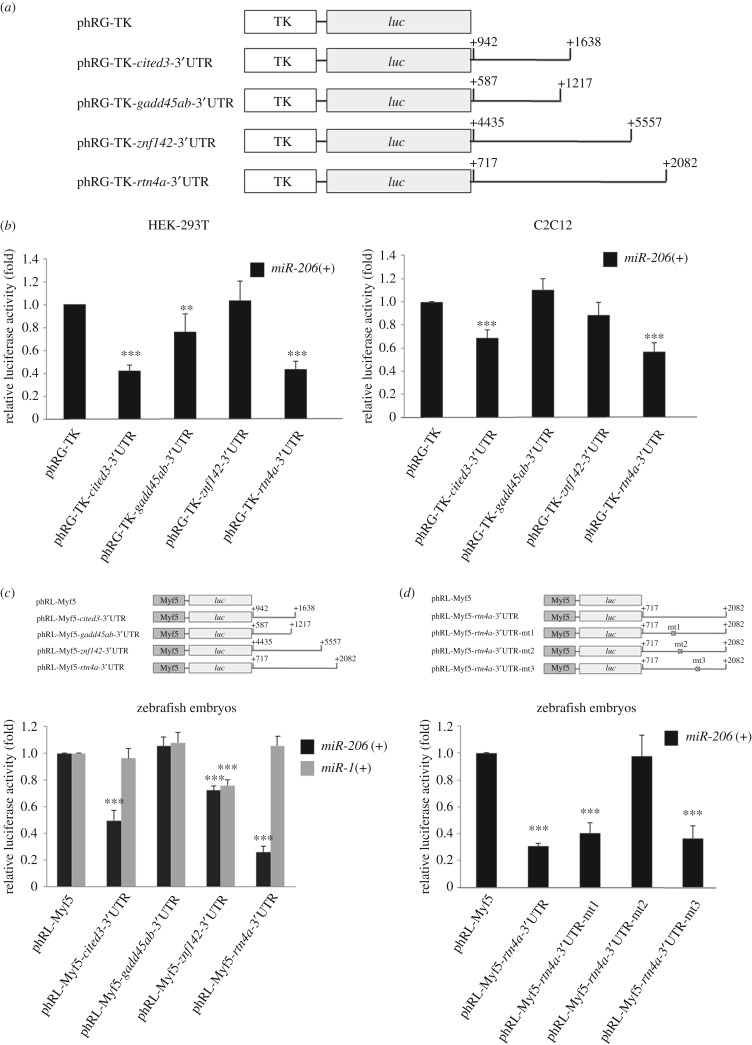
*miR-206* silences the expression of *luc* reporter through binding the 3′UTR from *rtn4a.* (*a*) Constructs for examining the *luc* assay. The complete 3′UTR segments of *cited3*, *gadd45ab*, *znf142* and *rtn4a,* which are four putative target genes for *miR-206*, were individually ligated into the downstream of the *luc* reporter gene and driven by thymidine kinase (TK) promoter in plasmid phRG-TK. (*b*) For *in vitro* study, plasmid pCS2-*miR-206* (indicated as *miR-206*(+)) was co-transfected with either pGL3-TK (internal control) or each examined construct, as indicated, into HEK-293T and C2C12 cells. Luciferase (*luc*) activity of each group was quantified, and its relative *luc* activity presented in fold was calculated based on the *luc* activity obtained from pCS2-*miR-206* combined with phRG-TK normalized as 1. (*c*) For *in vivo* study, either synthetic pre-*miR-206* RNA (*miR-206*(+)) or pre-*miR-1* RNA (*miR-1*(+)), in combination with plasmid phRL-Myf5 and each examined construct, was injected into zebrafish embryos. Plasmid phRL-Myf5 served as a control, in which *luc* expression was driven by the *myf5* promoter, and its *luc* activity was normalized as 1. The complete 3′UTR segments of *cited3, gadd43ab, znf142* and *rtn4a* were individually engineered into the downstream of the *luc* reporter gene and driven by the *myf5* promoter in plasmid phRL-Myf5. (*d*) Mutated sequences (mt1, mt2 and mt3; see Material and methods) of *rtn4a*-3′UTR were separately fused downstream of the *luc* reporter gene and driven by the *myf5* promoter to construct plasmids as indicated. The *luc* activity obtained from co-injection of synthetic pre-*miR-206* RNA (*miR-206*(+)) combined with plasmid phRL-Myf5 in embryos was normalized as 1. Each plasmid plus *miR-206* was individually injected in zebrafish embryos to performte *luc* assay. Data were presented as mean ± s.d. from three independent experiments (*n* = 3). Cross-filled box: *miR-206*-target mutated sequences on *rtn4a*-3′UTR. Asterisks indicate the significant difference level at **p < 0.01 and ***p < 0.001.

To further confirm whether *cited3*, *gadd45ab*, *znf142* and *rtn4a* were the target gene(s) of *miR-206*, we cloned their 3′ UTRs and fused them downstream of reporter cDNA encoding *Renilla* *luc* and driven by herpes simplex virus thymidine kinase promoter (TK). Thus, four expression plasmids were constructed: phRG-TK-*cited3*-3′UTR, phRG-TK-*gadd45ab*-3′UTR, phRG-TK-*znf142*-3′UTR and phRG-TK-*rtn4a*-3′UTR (figure 1*a*). These constructs were separately co-transfected with pCS2-*miR-206* into cell lines HEK-293T and C2C12 (figure 1*b*). Compared with the *luc* activity from the phRG-TK group (control) which was normalized as 1, the *luc* activities of HEK-293T cells transfected with phRG-TK-*cited3*-3′UTR, -*gadd45ab*-3′UTR, -*znf142*-3′UTR and -*rtn4a*-3′UTR were 0.42 ± 0.05, 0.76 ± 0.16, 1.04 ± 0.17 and 0.44 ± 0.07, respectively, while in the C2C12 cells they were 0.69 ± 0.07, 1.10 ± 0.09, 0.88 ± 0.11 and 0.57 ± 0.07, respectively (figure 1*b*). Since *luc* activity was greatly inhibited by *miR-206* in both non-muscle and muscle cell lines transfected with phRG-TK-*cited3*-3′UTR and phRG-TK-*rtn4a*-3′UTR, we chose only *cited3* and *rtn4a* for further *in vivo* experiments.

For the *in vivo* assay, we constructed phRL-Myf5-*cited3*-3′UTR, phRL-Myf5-*gadd45ab*-3′UTR, PhRL-Myf5-*znf142*-3′UTR and phRL-Myf5-*rtn4a*-3′UTR (figure 1*c*), in which the *luc* reporter was driven by zebrafish *myf5* promoter, a somite-specific promoter [33]. These constructs were co-injected with either pre-*miR-1* RNA or pre-*miR-206* RNA into one-cell zebrafish embryos. Compared with the *luc* activity of control embryos injected with phRL-Myf5 alone, which was normalized as 1, the *luc* activities of embryos injected with pre-*miR-206* RNA combined with plasmids phRL-Myf5-*cited3*-3′UTR, phRL-Myf5-*gadd45ab*-3′UTR, phRL-Myf5-*znf142-*3′UTR and phRL-Myf5-*rtn4a*-3′UTR were 0.49 ± 0.09, 1.05 ± 0.06, 0.72 ± 0.02 and 0.26 ± 0.03, respectively (figure 1*c*). On the other hand, the *luc* activities of embryos injected with pre-*miR-1* RNA combined with plasmids phRL-Myf5-*cited3*-3′UTR, phRL-Myf5-*gadd45ab*-3′UTR, PhRL-Myf5-*znf142*-3′UTR and phRL-Myf5-*rtn4a*-3′UTR were 0.96 ± 0.07, 1.08 ± 0.07, 0.75 ± 0.04 and 1.05 ± 0.07, respectively (figure 1*c*). This evidence indicated that *miR-206* can only specifically silence the reporter gene through *cited3*- and *rtn4a*-3′UTR, even though *miR-1* and *miR-206* have identical seed sequences. However, because *rtn4a*-3′UTR showed more obvious inhibition by *miR-206,* we focused on target gene *rtn4a* for further study.

### 3.2. *miR-206* was unable to silence reporter gene expression driven by mutated 3′UTR of *rtn4a*

The FindTar, RNA22 and RNAhybrid software programs were used to analyse the 3′UTR of zebrafish *rtn4a*, and three putative binding sequences for *miR-206* in *rtn4a*-3′UTR were found. We therefore mutated the nucleotides at these positions and constructed plasmids phRL-Myf5-*rtn4a*-3′UTR-mt1, -mt2 and -mt3, in which 1353 ∼ 1379 nt, 1422 ∼ 1444 nt and 1554 ∼ 1576 nt were mutated, respectively (figure 1*d*). Compared with the *luc* activity of embryos injected with pre-*miR-206* plus phRL-Myf5, which was normalized as 1, the *luc* activities of embryos injected with pre-*miR-206* RNA combined with phRL-Myf5-*rtn4a*-3′UTR, -*rtn4a*-3′UTR-m1, -*rtn4a*-3′UTR-mt2 and -*rtn4a*-3′UTR-mt3 were 0.31 ± 0.02, 0.40 ± 0.08, 0.97 ± 0.16 and 0.36 ± 0.09, respectively (figure 1*d*). This evidence indicated that injection of phRL-Myf5-*rtn4a*-3′UTR-mt2 abolishes the silencing effect of *miR-206.* Taken together, it was plausible to conclude that *miR-206* silences the translation of reporter gene through binding *rtn4a*-3′UTR at 1422 ∼ 1444 nt.

### 3.3. Overexpression of each isoform of Rtn4a causes abnormally transverse actin filaments in somites

**Figure 2.**
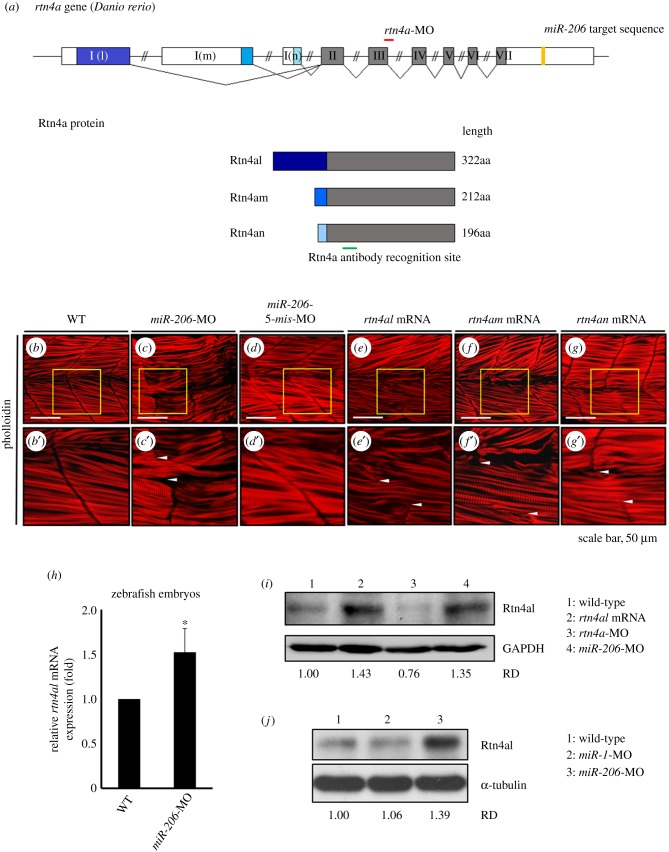
Knockdown of endogenous *miR-206* increases the level of Rtn4al protein, causing abnormal transverse actin filaments across the somite boundary. (*a*) The genomic structure of three isoforms of *rtn4a* genes. Locations of the *rtn4a*-MO binding sequence (red line), *miR-206* binding site at the 3′UTR of *rtn4a* mRNA (yellow box), and recognized region of antibody against Rtn4a protein (green line) were indicated. (*b*) WT, (*c*) *miR-206*-MO-injected, (*d*) *miR-206*-5-mis-MO-injected, (*e*) *rtn4al-*mRNA-injected, (*f*) *rtn4am-*mRNA-injected, and (*g*) *rtn4an-*mRNA-injected embryos developed at 48 hpf were immunostained with fluorescent phalloidin to label F-actin. Panels (*b*′*–g*′) were the enlarged views of corresponding panels (*b–g*). The area between the two arrowheads indicates the abnormal transverse actin filaments across the somite boundary. (*h*) The relative amounts of *rtn4al* mRNA between WT deheaded embryos and *miR-206*-MO-injected deheaded embryos at 20 hpf were quantified by qPCR when that of WT was normalized as 1. One hundred embryos were used each time, and experiments were performed in triplicate (*n* = 3). (*i*) Western blot analysis of Rtn4al (35 kD) among proteins extracted from deheaded embryos at 20 hpf: in WT (lane 1), *rtn4al*-mRNA-injected embryos (lane 2), *rtn4a*-MO-injected embryos (lane 3) and *miR-206*-MO-injected embryos (lane 4). (*j*) Western blot analysis of Rtn4al among proteins extracted from deheaded embryos at 20 hpf: in WT (lane 1), *miR-1*-MO-injected embryos (lane 2), and *miR-206*-MO-injected embryos (lane 3). Student's *t*-test was used for statistical analysis. Asterisk indicates significant difference at *p < 0.05. RD: Relative density. GAPDH and tubulin served as internal controls.

Three *rtn4a* isoforms are found in zebrafish, named *rtn4al*, *rtn4am* and *rtn4an* [37,38]. They share identical C-terminal sequence and 3′UTR, including 1422 ∼ 1444 nt, which was bound by *miR-206* in the experiment described above (figure 2*a*). In order to exclude the off-target effect of MO injection, we knocked down endogenous *miR-206* by injection of *miR-206*-MO which specifically inhibits both *miR-206-1* and *miR-206-2* in zebrafish embryos without affecting the production of *miR-1* with the same seed sequence as that of *miR-206* [5]. As control-MO, we used *miR-206*-*5-mis*-MO, as described previously [5]. When we injected mRNAs to individually overexpress *rtn4al*, *rtn4am* or *rtn4an* in the zebrafish embryos, defective phenotypes exhibiting abnormal transverse actin filaments across the somite boundary could be observed at 48 hpf in embryos injected as noted above (figure 2*b–g*), except the control-MO injection group. By contrast, the A-band within actin filaments was arranged with no apparent change in order (figure 2*b*′*–g*′). Therefore, based on the data obtained from the rescue experiment, as shown in the electronic supplementary material, table S2, it was concluded that either knockdown of *miR-206* or overexpression of each isoform of Rtn4a in embryos causes the observed defective phenotype. These somite boundary formation defects can be rescued either by overexpression of mature *miR-206* RNA or knockdown of *rtn4a*. Thus we suggested that either knockdown of *miR-206* or overexpression of each isoform of Rtn4a caused the observed defective phenotype, but did not disturb the arrangement of actin or muscle fibre development.

Since overexpression of each isoform of Rtn4a caused defective actin filaments in somites, we focused on *rtn4al* for further study. Using WISH, we found that *miR-206* was detectable in somites and PSM as early as at 12 hpf, while *rtn4al* was detectable in somites at 16 hpf (electronic supplementary material, figure S1). Furthermore, using frozen sections, we observed that both *miR-206* and *rtn4al* were expressed in the fast muscle of trunk at 24 hpf (electronic supplementary material, figure S1), suggesting a tight, possibly regulatory, relationship between *miR-206* and *rtn4al* in the zebrafish somite.

### 3.4. Knockdown of *miR-206* increases *rtn4al* mRNA and Rtn4al protein in zebrafish embryos

Using qPCR, we quantified *rtn4al* mRNA expression level in zebrafish embryos injected with *miR-206*-MO to specifically knock down endogenous *miR-206*. The amount of *rtn4al* mRNA in the untreated WT embryos at 20 hpf was normalized as 1, and the amount of *rtn4al* mRNA in embryos injected with *miR-206*-MO was 1.52 ± 0.31 (*n* = 3) (figure 2*h*), which represents an approximately 52% increase. This qPCR result was consistent with WISH detection in embryonic somites, which demonstrated that the *rtn4al* mRNA signal shown in *miR-206*-MO-injected embryos was stronger than that of WT (electronic supplementary material, figure S2). Furthermore, compared with WT, we found that the protein level of Rtn4al was increased in the *miR-206*-MO-injected embryos at 20 hpf (figure 2*i*). However, the protein level of Rtn4al remained unchanged in the embryos injected with *miR-1*-MO (figure 2*j*, lane 2), which shares an identical seed sequences with *miR-206* and whose antisense oligonucleotide sequence were previously described by Lin *et al.* [5], indicating that the amount of Rtn4al protein is specifically regulated by *miR-206*, not *miR-1*, thus confirming our speculation.

### 3.5. Either knockdown of *miR-206* or overexpression of *rtn4al* causes defective somite boundary in embryos

**Figure 3.**
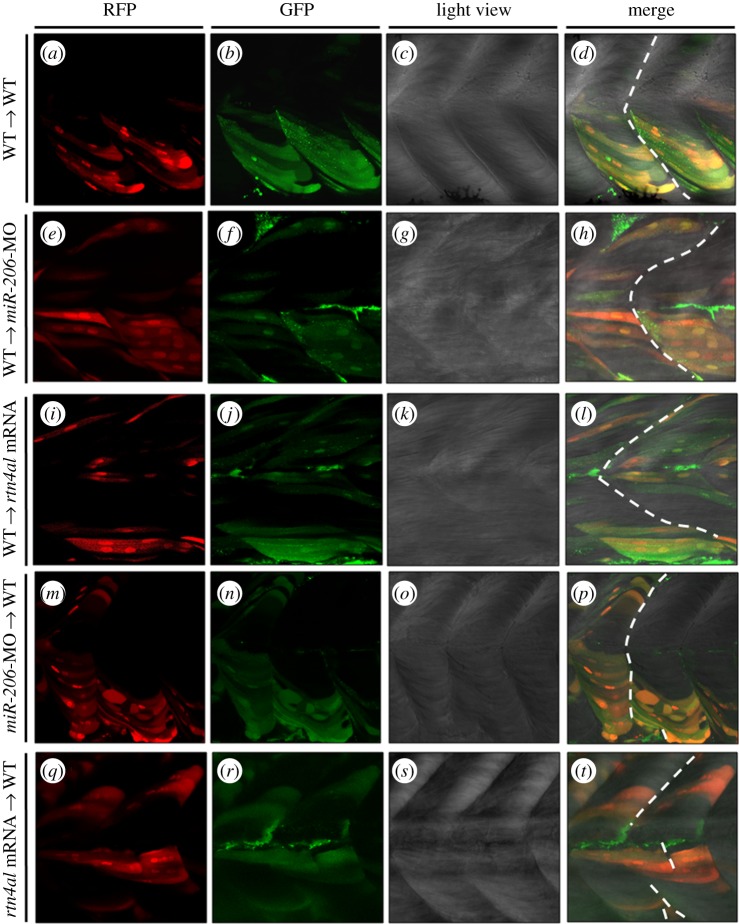
Transplantation of cells derived from either *miR-206*-MO- or *rtn4al*-mRNA-injected embryos causes recipient embryos to generate somite boundary defects. (*a–d*) After Dextran was injected into *Tg(α-actin:RFP)* embryos at the one-cell stage, cells were taken from donor embryos at 4 hpf and transplanted into WT embryos at 4.7 hpf. Embryonic somite boundary development was observed when embryos were 48 hpf. (*a*) Differentiated muscle cells among the transplanted cells were marked by RFP. (*b*) All transplanted cells were marked with green fluorescent signal. (*c*) Somite morphology was observed under microscopic light field. (*d*) Somite boundary was normally formed. (*e–h*) Transplanted cells from Dextran-injected *Tg(α-actin:RFP)* embryos to *miR-206*-MO-injected embryos. (*i–l*) Transplanted cells from Dextran-injected *Tg(α-actin:RFP)* embryos to *rtn4al-*mRNA-injected embryos. (*m–p*) Transplanted cells from Dextran- and *miR-206*-MO-injected *Tg(α-actin:RFP)* embryos to WT embryos. (*q–t*) Transplanted cells from Dextran- and *rtn4al-*mRNA-injected *Tg(α-actin:RFP)* embryos to WT embryos. Broken line indicates formation of the somite boundary.

To determine if any change of *miR-206* and its target gene might cause a corresponding defect in embryos, we employed zebrafish transgenic line *Tg(α-actin:RFP)*, in which muscle cells are tagged with red fluorescent protein (RFP) [30]. Embryos classified as donor groups received injection of the green fluorescent dye Dextran alone or injection of Dextran combined with either *miR-206*-MO or *rtn4al* mRNA in *Tg(α-actin:RFP)*. After *Tg(α-actin:RFP)* embryos were treated and developed at 4 hpf, 20–30 cells were taken from donor embryos and transplanted into the non-axial mesoderm of recipient (WT or MO/mRNA-injected) embryos at 4.7 hpf. The control group showed no somite boundary defect in recipient embryos (figure 3*a–d*) (*n* = 15). Additionally, when cells from WT embryos were transplanted into either *miR-206*-MO- (figure 3*e–h*) (*n* = 20) or *rtn4al*-mRNA-injected recipients (figure 3*i–l*) (*n* = 18), no somite boundary defect was observed. However, somite boundary of recipient embryos transplanted with cells from embryos injected with either Dextran combined with *miR-206*-MO (figure 3*m–p*) (*n* = 21) or *rtn4al* mRNA (figure 3*q–t*) (*n* = 24) appeared defective (ectopic or loss) by 23.8 and 33.3%, respectively, indicating that the number of somite boundary defects caused by knockdown of *miR-206* and overexpression of Rtn4al had increased. This quantitation experiment strongly suggests that either knockdown of *miR-206* or overexpression of Rtn4al results in defective formation of the somite boundary, indicating it is an example of a community effect.

Based on these findings, we asked if such defect resulted from abnormal regulatory factors involved in somite boundary formation, such as *fgf8*, *deltad*, *her1*, *tbx6*, *mespa* and *mespb.* As shown in the electronic supplementary material, figure S3, the expression patterns of these segmentation decision genes were not significantly altered in either *miR-206-*knockdown or Rtn4al-overexpression embryos. These data suggested that the effect of the *miR-206/rtn4al* axis on somite boundary formation is independent of the effect on somite formation, giving reason to hypothesize that it might be mediated through some unknown downstream effectors.

### 3.6. Genes that were predominantly impacted by both *miR-206-*knockdown and *rtn4al*-overexpression in the somite of embryos

To discover potential regulatory factors mediating the effect of *miR-206/rtn4al* axis in somites, we employed another transgenic line, *Tg(myf5* : *GFP)*, in which the GFP reporter is driven by an upstream 80 kb of zebrafish *myf5* such that GFP is expressed in the developing PSM and somite [29]. After we injected *miR-206*-MO and *rtn4al* mRNAs separately into *Tg*(*myf5:GFP*) embryos, cells were dissociated from embryos at 20 hpf. We collected the GFP-expressing cells through fluorescence-activated cell sorting (FACS) and then performed microarray. We found 33 and 60 genes greatly increased and decreased, respectively, in both *miR-206-*knockdown embryos and the *rtn4al*-overexpression embryos compared with the untreated embryos (electronic supplementary material, figure S4). From these genes, we selected four upregulated genes, including *DiGeorge syndrome chromosomal region 8* (*dgcr8*), *protein O-mannosyl-transferase 1* (*pomt1*), *NFKB inhibitor interacting Ras-like 2* (*nkiras2*) and *thbs3a*, and four downregulated genes, including *zgc 56251*, *Sal-like protein 4* (*sall4*), *cxcr4a* and *syndecan 4* (*sdc4*), and used WISH to determine their proportional expression in somites relative to the results from microarray (electronic supplementary material, figure S5). On the basis of WISH results, we found that *thbs3a* and *cxcr4a* were highly expressed in the somite boundary region, and these two genes were chosen for further study.

### 3.7. Somite boundary formation defect caused by abnormal expression of *cxcr4a* and *thbs3a* is similar to that caused by abnormal expression of *miR-206* and *rtn4al*

**Figure 4.**
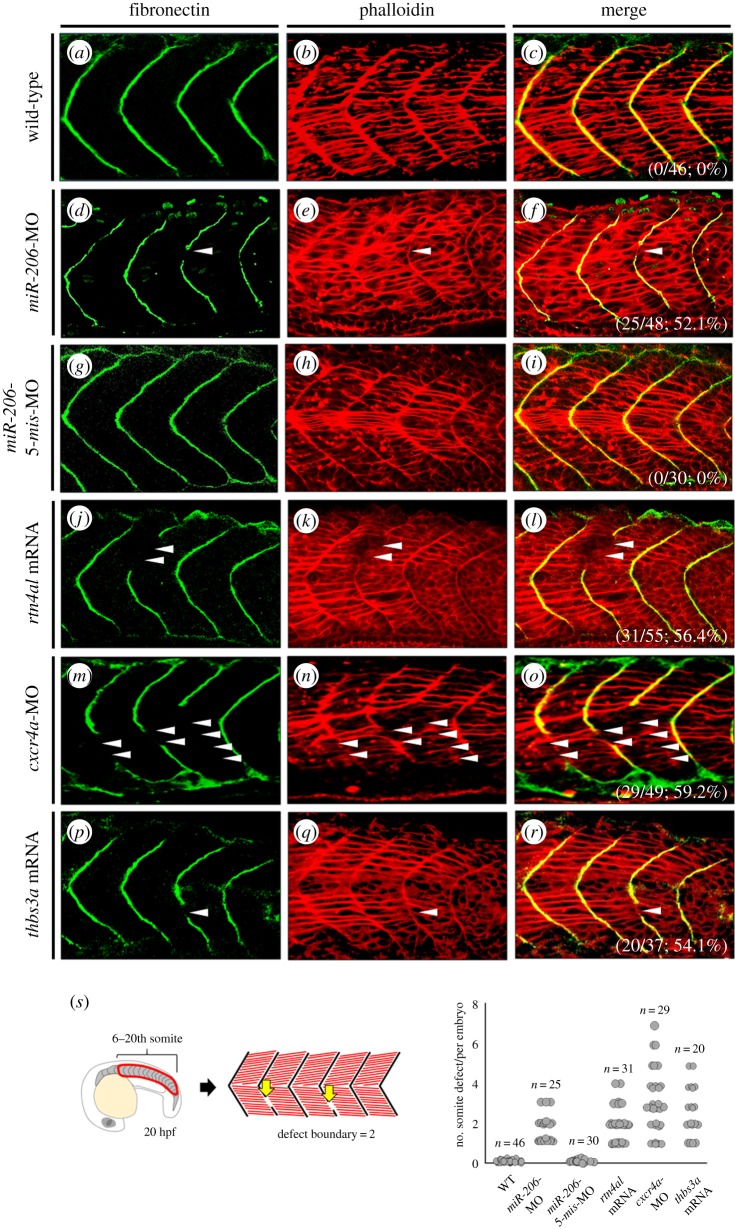
Somite boundary defect occurred in either *cxcr4a*-knockdown or *thbs3a*-overexpression zebrafish embryos. (*a–c*) None treated control WT embryos, (*d–f*) *miR-206-*knockdown embryos, (*g–i*) *miR-206-5-mis*-MO-injected embryos (served as control), (*j–l*) *rtn4al-*mRNA-overexpressed embryos, (*m–o*) *cxcr4a-*knockdown embryos, and (*p–r*) *thbs3a-*mRNA-overexpressed embryos were examined. Immunofluorescent staining was performed on the embryos at 20 hpf. (*a*,*d*,*g*,*j*,*m*,*p*) Fibronectin was labelled with green fluorescent signal to detect somite boundary. (*b*,*e*,*h*,*k*,*n*,*q*) Phalloidin was labelled with red fluorescent signal to detect F-actin. (*c*,*f*,*i*,*l*,*o*,*r*) Merge of the two signals. Places where the somite boundary was absent were marked by white arrowheads. The numbers shown on the lower-right corner are the percentages of defective somite boundary occurrence averaged from three independent experiments. (*s*) Quantification of the number of defective boundaries per embryo at the 6th to 20th pairs of somites on both sides of the trunk at 20 hpf.

The somite boundary was normally developed in WT embryos at 20 hpf (figure 4*a–c*), while was incompletely formed in embryos injected with *miR-206*-MO (figure 4*d–f*), *miR-206*-*5-mis*-MO (figure 4*g–i*), *rtn4al* mRNA (figure 4*j–l*), *cxcr4a*-MO (figure 4*m–o*) and *thbs3a* mRNA (figure 4*p–r*). The percentages of embryos exhibiting defective formation of the somite boundary among examined embryos were 0% (*n* = 46) in the WT group, 52.1% (*n* = 48) in the *miR-206*-MO-injection group, 0% (*n* = 30) in the *miR-206*-*5-mis*-MO-injection group, 56.4% (*n* = 55) in the *rtn4al*-mRNA-injection group, 59.2% (*n* = 49) in the *cxc4a*-MO-injected group, and 54.1% (*n* = 37) in the *thbs3a*-mRNA-injection group. We observed that defective somite boundary was randomly distributed along the trunk, even occurring on both sides of the trunk in zebrafish embryos. Therefore, to determine the disrupted boundary located at the 6th to 20th pairs of somites on both sides of the trunk in 20 hpf embryos, we quantified the number of embryos having this defective boundary among examined embryos. Additionally, we calculated the number of defective somites per embryo. As shown in figure 4*s*, we demonstrated that defective formation of somite boundary caused by abnormal expression of *cxcr4a* and *thbs3a* was completely congruent with that caused by abnormal expression of *miR-206* and *rtn4al.* Additionally, we found that this somite boundary defect did not result from developmental delay because this defect could still be observed in the injected embryo up to 48 hpf (electronic supplementary material, figure S6 and table S2). Although somite patterning and somite boundary formation genes, such as *fgf8, deltad, her1, tbx6, mespa,* and *mespb*, were not significantly different from those of the control group (electronic supplementary material, figure S3), we observed that the ECM, consisting of fibronectin and laminin, was not correctly organized in the somite boundary of embryos at 20 hpf (figure 4) and 48 hpf (electronic supplementary material, figure S6; table S2) when embryos were injected with either *miR-206*-MO or *rtn4al* mRNA. These results were similar to those reported by Goody *et al.* [39] for *nrk2b*-MO-injected embryos. Although somites were able to form normally starting at 20 hpf, we noticed that somite boundary formation could be disrupted and fail to form completely by 48 hpf by the failed epithelialization of somite boundary cells, in turn resulting in discontinuous MTJ [39,40]. Thus, some muscle fibres were abnormally transverse across places where the somite boundary had incompletely formed. This evidence supports Henry *et al.* [40] who demonstrated that the disorganization of fibronectin directly impacted failed epithelialization of boundary cells.

### 3.8. Cxcr4a and Thbs3a are downstream effectors of the *miR-206/rtn4al* axis

**Figure 5.**
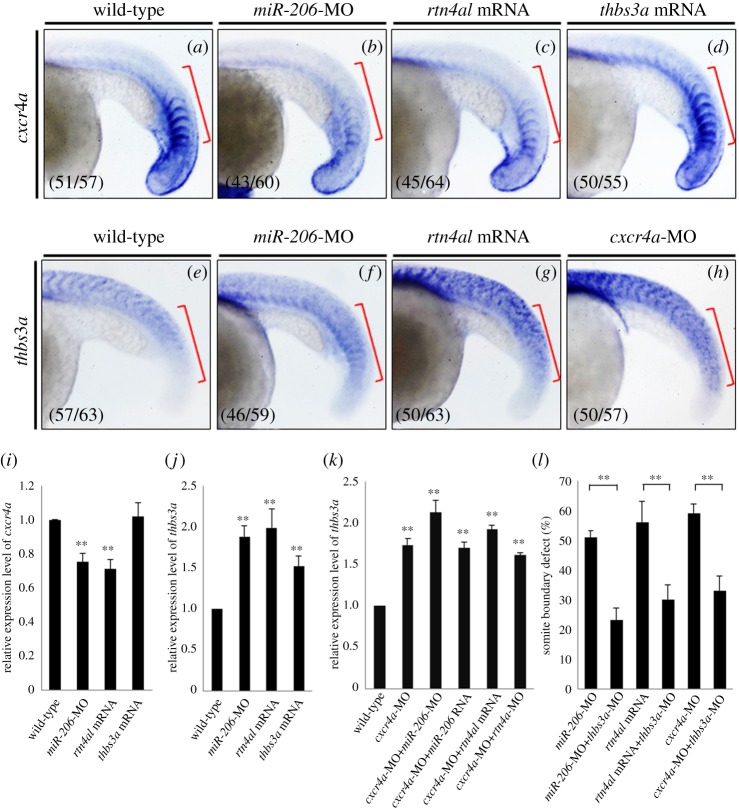
Injection of *cxcr4a* can repress *thbs3a* expression in zebrafish somites, but *thbs3a* cannot repress *cxcr4a*. Using WISH to detect the spatial expression patterns of (*a–d*) *cxcr4a* and (*e–h*) *thbs3a* in somites of zebrafish embryos at 20 hpf. (*a*,*e*) WT embryos; (*b*,*f*) knockdown of *miR-206*; (*c*,*g*) overexpression of Rtn4al; (*d*) overexpression of Thbs3a; and (*h*) knockdown of Cxcr4a. The *cxcr4a* expression level was reduced in the somites of (*b*) *miR-206*-MO-injected embryos and (*c*) *rtn4al*-mRNA-injected embryos, while (*d*) *cxcr4a* expression was not affected by overexpression of Thbs3a. On the other hand, *thbs3a* expression level was increased in the somites of (*f*) *miR-206*-MO-injected embryos and (*g*) *rtn4al* mRNA-injected embryos, while (*h*) *thbs3a* expression was also increased in *cxcr4a*-MO-injected embryos. Data shown at the lower-left corner are the number of phenotypes out of the examined embryos. (*i*,*j*) The expression levels of *cxcr4a* and *thbs3a* in each group were quantified. (*k*) Using q-PCR to quantify the levels of *thbs3a* mRNA expression in WT and embryos injected either *cxcr4a*-MO alone or *cxcr4a*-MO combined with *miR-206*-MO, *miR-206* RNA, *rtn4al*-MO or *rtn4al* mRNA. One hundred embryos were studied each time, and three independent experiments were performed (*n* = 3). (*l*) *miR-206*-MO-, *rtn4al*-mRNA-, or *cxcr4a*-MO-injected embryos together with knockdown of *thbs3a* all reduced the percentages of defective boundary. Numbers shown at the lower-left corner were the numbers of phenotypes out of the examined embryos. Student's *t*-test was used for statistical analysis. Asterisks indicate the significant difference level at **p < 0.01.

To further confirm whether Cxcr4a and Thbs3a are downstream effectors of the *miR-206/rtn4al* axis, we individually injected *miR-206*-MO, *rtn4al* mRNA and *thbs3a* mRNA into one-cell zebrafish embryos, followed by detection of the expression level of *cxcr4a* mRNA at 20 hpf. In the WT embryos, we found that *cxcr4a* displayed a pronounced expression in PSM and boundaries in the newly forming somites, but only weak expression in mature somites (figure 5*a*). In the *miR-206*-knockdown and *rtn4al*-overexpression embryos, *cxcr4a* was reduced in newly forming somites (figure 5*b*,*c*), suggesting that *crcx4a* is negatively regulated by *rtn4a*. However, *cxcr4a* remained unchanged in *thbs3a*-overexpression embryos (figure 5*d*), suggesting that *cxcr4a* is not regulated by *thbs3a*. These results observed from the WISH were consistent with the data obtained from q-PCR (figure 5*i*).

On the other hand, we individually injected *miR-206*-MO, *rtn4al* mRNA and *cxcr4a-*MO into one-cell zebrafish embryos, followed by detection of the expression level of *thbs3a* mRNA at 20 hpf. In the WT embryos, *thbs3a* was expressed at low level in the newly forming somites, but it was expressed at a relatively high level in mature somites (figure 5*e*). In the *miR-206-*knockdown*,* *rtn4al*-overexpression and *cxcr4a-*knockdown embryos, however, *thbs3a* was expressed at a relatively high level in the newly forming somites with pronounced expression in mature somites (figure 5*f–h*). Again, these WISH results were consistent with the data obtained from qPCR (figure 5*j*). Furthermore, we also employed qPCR to quantify the level of *thbs3a* mRNA expression in embryos injected either *cxcr4a*-MO alone or *cxcr4a*-MO combined with *miR-206*-MO, *miR-206* RNA, *rtn4al*-MO or *rtn4al* mRNA. As shown in figure 5*k,* when *cxcr4a* was knocked down in embryos, *thbs3a* expression was increased, irrespective of whether *miR-206* or *rtn4al* was increased or decreased. This line of evidence suggested that *miR-206* and *rtn4al* do not regulate *thbs3a* expression in *cxcr4a* morphants. However, *cxcr4a* is an upstream negative regulator of the *thbs3a* gene*.* Therefore, Cxcr4a has a direct and significant effect on Thbs3a. Additionally, injection of *miR-206*-MO, *rtn4al* mRNA or *cxcr4a-*MO combined with *thbs3a*-MO in embryos resulted in the reduction of *thbs3a* expression. Interestingly, we demonstrated that the somite boundary formation defect caused by *miR-206-*knockdown*, rtn4al*-overexpression and *cxcr4a*-knockdown could be rescued by reduction of Thbs3a (figure 5*l*).

Based on this line of evidence, we concluded that Cxcr4a is an upstream negative regulator controlling *thbs3a* expression in newly formed somites, while Rtn4al is an upstream negative regulator controlling *cxcr4a* expression. Meanwhile, *miR-206* is present in PSM and somites (electronic electronic supplementary material, figure S2), and it is able to repress Rtn4al, resulting in the higher expression of Cxcr4a, which, in turn, prevents excessive expression of Thbs3a in newly forming somites, leading to normal formation of somite boundaries during somitogenesis. These findings allowed us to propose a novel *miR-206/rtn4a/cxcr4a/thbs3a* regulatory cascade that mediates the formation of normal somite boundary.

### 3.9. The *miR-206/rtn4a/cxcr4a/thbs3a* cascade plays a role in the MET of epithelial cells to form thesomite boundary

**Figure 6.**
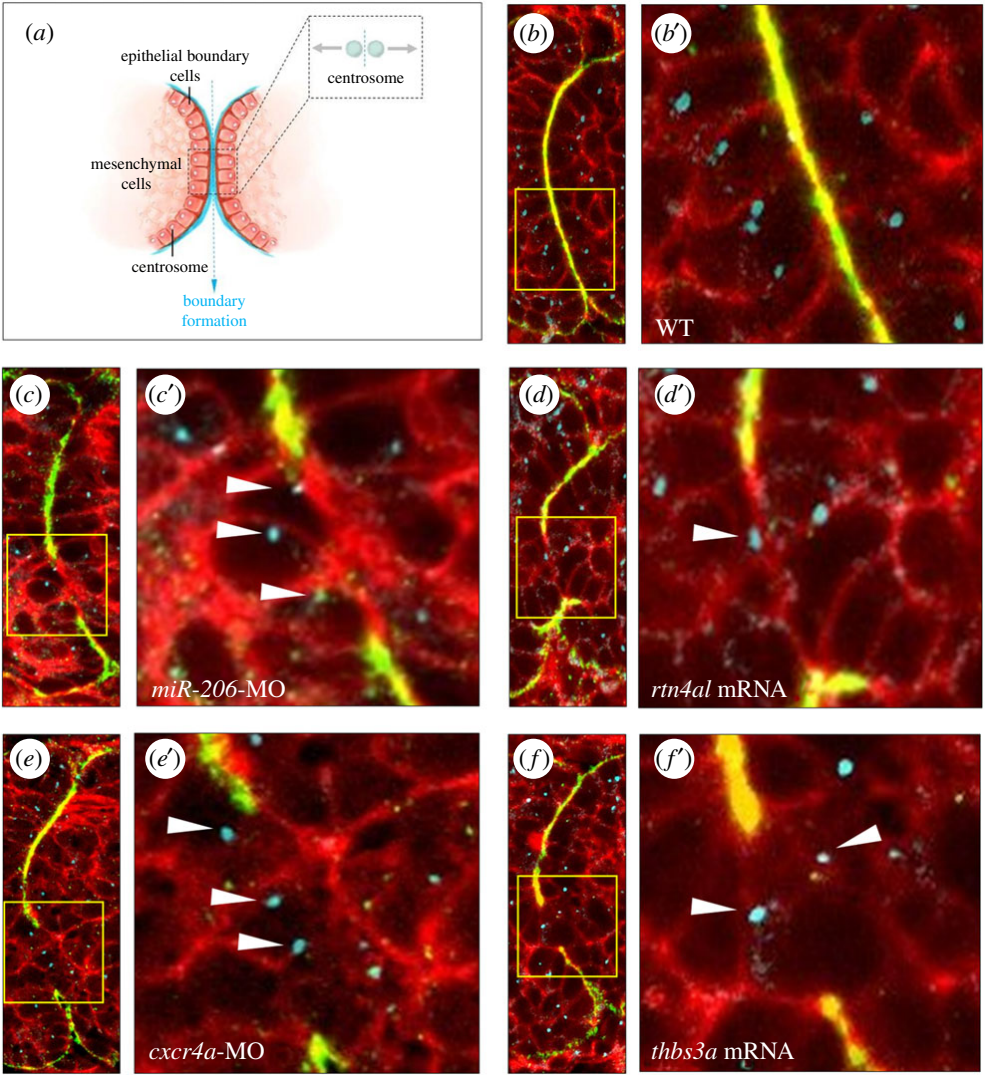
Change of expression levels of *miR-206*, Rtn4al, Cxcr4a or Thbs3a fails to epithelialize somites in zebrafish embryos. (*a*) A diagram depicts that centrosomes of epithelial cells at the somite boundary are localized apically when epithelial cells undergo MET. (*b*) WT embryos at 20 hpf; (*c*) knockdown of *miR-206*, (*d*) overexpression of *rtn4al*, (*e*) knockdown of *cxcr4a* and (*f*) overexpression of *thbs3a.* Fibronectin labelled with green fluorescent signal was used to mark the somite boundary; Phalloidin labelled with red fluorescent signal was used to mark F-actin, while *γ*-tubulin labelled with blue fluorescent signal was used to mark centrosomes. (*b–f*) Three fluorescent signals were merged; (*b*′*–f*′) were amplified from the corresponding panels (*b–f*). White arrowheads indicate centrosomes not localized apically in the epithelial cells of the defective somite boundary.

**Figure 7.**
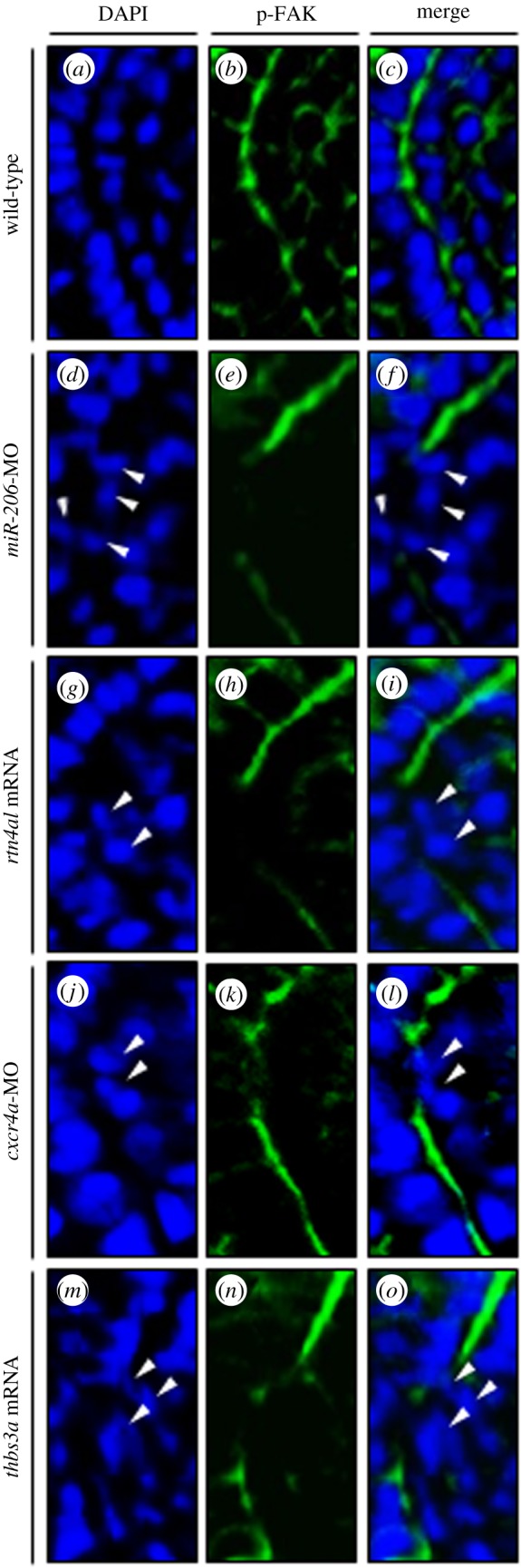
Change of the expression levels of *miR-206*, Rtn4al, Cxcr4a and Thbs3a resulted in the failure of p-FAK to concentrate at the intersomitic boundary of defective boundary cells. (*a–c*) In the WT embryos at 20 hpf, p-FAK signal was concentrated at the position of the intersomitic boundary; (*d–f*) knockdown of *miR-206*, (*g–i*) overexpression of *rtn4al*, (*j–l*) knockdown of *cxcr4a* and (*m–o*) overexpression of *thbs3a*. DAPI labelled with blue fluorescent signal was used to mark the nucleus, while green fluorescent signal was used to label p-FAK. (*c*,*f*,*i*,*l*,*o*) Two fluorescent signals were merged. White arrowheads indicate that p-FAK did not concentrate at the intersomitic boundary of defective boundary cells.

During somite boundary formation, the epithelial cells of the somite boundary become columnar in shape, undergo MET, and exhibit cell polarity which makes centrosomes localize apically [23,36,41]. Using fluorescence-labelled γ-tubulin, we could trace the location of centrosomes in the epithelial cells of the somite boundary. In WT embryos, results showed that the epithelial cells of the boundary presented an oval or cylindrical shape and that centrosomes localized apically (figure 6*a*,*b*,*b*′). However, in the *miR-206*-MO-injected embryos (figure 6*c*,*c*′), *rtn4al*-mRNA-injected embryos (figure 6*d*,*d*′), *cxcr4a-*MO-injected embryos (figure 6*e*,*e*′) or *thbs3a*-mRNA-injected embryos (figure 6*f*,*f*′), the epithelial cells of the somite boundary presented a circular shape, and centrosomes did not localize apically (figure 6*b*′*–f*′, arrowhead). Instead, centrosomes were localized randomly. This line of evidence suggested that the epithelial cells of *miR-206*-knockdown embryos, *rtn4al*-overexpression embryos, *cxcr4a-*knockdown embryos and *thbs3a-*overexpression embryos do not undergo epithelialization at somite boundaries.

p-FAK is present in multiple receptor complexes and is located at the intersomitic boundary [42,43]. It is required for somite boundary formation during somitogenesis of zebrafish embryos [42]. Therefore, we detected the distribution pattern of p-FAK to confirm its concentration at the intersomitic boundary of embryos injected with *miR-206*-MO and *rtn4al* mRNA, and results showed the absence of intracellular accumulation of p-FAK in boundary cells. Thus, we detected p-FAK signal in WT embryos and embryos injected with *miR-206*-MO, *rtn4al-*mRNA, *cxcr4a*-MO and *thbs3a-*mRNA. In WT embryos, results showed that the p-FAK signal did not exhibit evenly in the entire boundary cells. Instead, p-FAK presented a high concentration toward the intersomitic position (figure 7*a–c*), as described previously by Crawford *et al.* [44]. However, unlike WT embryos, in the embryos injected with *miR-206*-MO (figure 7*d–f*), *rtn4al* mRNA (figure 7*g–i*), *cxcr4a-*MO (figure 7*j–l*) and *thbs3a* mRNA (figure 7*m–o*), the p-FAK signal did not present a high accumulation pattern towards the intersomitic position (white arrows), indicating that these defective boundary cells were unable to process epithelialization by the absence of intracellular p-FAK accumulation in the somite boundaries.

To test the hypothesis that increased Thbs3a causes decreased expression of p-FAK within cells, we further employed mesodermal C2C12 cells which were cultured in undifferentiated condition. When C2C12 cells were treated with overexpressed mouse Thbs3a (mThbs3a), the expression of intracellular p-FAK[pY397] was decreased (electronic supplementary material, figure S7*a*), suggesting that the result observed in the mThbs3a-overexpressed cells, namely decreased expression of intracellular p-FAK[pY397], was essentially replicated in the boundary cells of *thbs3a-*mRNA-injected embryos and, hence, the absence of intracellular p-FAK accumulation in the somite boundaries, explaining, in turn, the inability to process epithelialization. Additionally, we found that active Cdc42 was increased in mThbs3a-overexpressed cells (electronic supplementary material, figure S7*b*), indicating that these cells tend toward epithelial–mesenchymal transition (EMT), which is unfavourable for epithelialization.

## 4. Discussion

### 4.1. Novel *miR-206/rtn4a/cxcr4a/thbs3* pathway was found in newly forming somites to maintain and stabilize somite boundary formation

Skeletal muscle-specific *miR-206* is known to regulate the differentiation of muscle cells [45]. Additionally, *miR-206* represses the translation of mRNA encoding vascular endothelial growth factor Aa, resulting in inhibiting the angiogenesis of zebrafish embryos at 24–72 hpf [5,46]. Interestingly, in this study we reveal another biological function of *miR-206* during embryogenesis, namely that *miR-206* can repress the expression of *rtn4a* and thus play a role in the formation of the somite boundary during somitogenesis of zebrafish embryos at 16–20 hpf. Both *miR-1* and *miR-206* are known to share common expression in the skeletal muscle of organisms, ranging from *Caenorhabditis elegans* to humans [47,48]. They also share identical seed sequences within a 22-nt length of mature miRNAs [49]. However, we further found that Rtn4a expression is specifically inhibited by *miR-206,* not *miR-1*. Based on this evidence, we concluded that the regulatory pathway related to somite boundary formation is *miR-206*-specific, which is strongly supported by the hypothesis proposed by Lin *et al.* [5], who demonstrated that *miR-1* and *miR-206* target different genes and play different roles during zebrafish embryogenesis.

The study of zebrafish Rtn4a has largely been confined to development of the nervous system [7,8]. Meanwhile, although *rtn4a* knockdown does not cause a significant defect of somite development, the effect of *rtn4a* overexpression on somite development has not been reported. Pinzón-Olejua *et al.* [8] reported that the expression pattern of Rtn4a in zebrafish is noticeably present at the somite boundary, but the biological implication of Rtn4a in the somite boundary is still unknown. In this study, we demonstrated that Rtn4a plays a key role in somite boundary formation during somitogenesis. The reduced expression of Rtn4a, as mediated by *miR-206*, increased the expression of downstream *cxcr4a*, a gene well known to be involved in somite boundary formation [28], particularly in the newly forming somites of embryonic trunk. Our further study indicated that Cxcr4a represses the expression of downstream Thbs3a, an ECM protein. The reduced expression of Thbs3a favours epithelialization of boundary epithelium cells through MET to form boundaries in the newly forming somites. Therefore, we propose a novel regulatory pathway, *miR-206/rtn4a/cxcr4a/thbs3a*, which modulates somite boundary formation in zebrafish embryos.

We noticed that the expression levels among *rtn4a, cxcr4a* and *thbs3a* in the normal state are different between newly forming somites and mature somites during somitogenesis. As shown in figure 5, when we used WISH to examine the expression level of embryos at 20 hpf, we found that a relatively lower level of *rtn4a* was present in newly forming somites, which allowed a greater expression of *crcx4a*, but a decreased persistence of *thbs3a*, finally allowing boundary cells to undergo MET and form normal boundaries in the newly forming somites of zebrafish embryos. Unlike the expression levels that occurred in newly forming somites, a relatively higher level of *rtn4a* was present in mature somites, which downregulated the *cxcr4a* expression, resulting in the upregulation of *thbs3a*. However, the biological implication of increased Thbs3a in mature somites during somitogenesis requires further investigation; it is well outside scope of the present study.

### 4.2. Structurally defective myotendinous junction might cause muscle fibres to cross somite boundary

When zebrafish embryos were treated with knockdown of *miR-206,* overexpression of *rtn4a,* knockdown of *cxcr4a,* or overexpression of *thbs3a*, defective somite boundaries were observed at early (20 hpf) and late (48 hpf) developmental stages. However, at late stage, some elongated myofibres were frequently observed to cross the somite boundary. It was speculative that this phenomenon may have resulted from incomplete formation of MTJ, as explained below. The somite boundary in zebrafish embryos forms in three separate stages before 24 hpf [40]. The first stage involves formation of the initial epithelial somite boundary when epithelial border cells surround an inner mass of mesenchymal cells [11]. During the second transitional stage, mesenchymal cells start to differentiate into muscle cells. Myotome boundary formation occurs during the third and final stage when fibronectin and p-FAK accumulate at somite border cells [40]. In zebrafish muscle differentiation, the fibronectin-rich matrix concentrates adjacent to slow-twitch fibres, while the laminin-rich basement membrane concentrates adjacent to both slow-twitch and fast-twitch muscle fibres. Thereafter, MTJ forms in this ECM-rich area between muscle segments [22,50].

In *miR-206*-knockdown and *rtn4a*-overexpression embryos, we found that fibronectin and p-FAK did not accumulate correctly in the border cells of embryos as early as 20 hpf (figures 4 and 7). Additionally, their boundary cells did not properly process initial epithelial somite boundary formation at 20 hpf. Since laminin was then unable to accumulate correctly in the somite boundary at 48 hpf (electronic supplementary material, figure S6), MTJ was incompletely formed, as noted above. In this case, absence of any barrier between two somites, muscle cells differentiate and fuse into a long muscle fibre that crosses the compromised site of the somite boundary. However, whether there is any implication of the *miR-206/rtn4/crcx4a/thbs3a* axis reported in this study relative to MTJ formation at any particular stage in zebrafish embryos is an interesting issue for further study.

### 4.3. *miR-206* affects somite boundary formation by regulating MET

As a therapeutic agent, *miR-206* has been used to treat drug-resistant cells and cancer cells based on its ability to suppress EMT. For example, overexpression of *miR-206* was used to treat breast cancer cells to inhibit the downstream genes of *transforming growth factor* (*TGF*)-*β*, such as *NRP1* and *SMAD2*, to reduce the migration and invasion of breast cancer cells [51]. Overexpression of *miR-206* was also used to treat lung adenocarcinoma cisplatin-resistant cells to enhance MET protein level and, in turn, restrict the migration and invasion of lung cancer cells [52]. By contrast, knockdown of *miR-206* favours cells undergoing EMT. This evidence indicates that *miR-206* is involved in balancing the EMT/MET biological process. In this study, we observed that knockdown of *miR-206* affects somite boundary formation in embryonic development through disturbance of somite boundary epithelial cells undergoing MET. Therefore, the use of *miR-206* overexpression as a tumour suppressor through regulating EMT/MET supports our findings because *miR-206* overexpression favours MET which results in epithelialization of boundary cells to form normal boundary, while *miR-206* knockdown favours EMT which results in failure of epithelialization of boundary cells and forms a defective boundary.

### 4.4. *miR-206* does not directly affect the expressional changes of *cxcr4a* or *thbs3a*

We analysed two individual microarrays obtained from *miR-206*-knockdown embryos and *rtn4a*-overexpressed embryos. We found that the expressions of downstream *cxcr4a* and *thbs3a* were consistent in that *cxcr4a* was decreased and *thbs3a* was increased in both microarrays (electronic supplementary material, figure S5). However, neither *cxcr4a* nor *thbs3a* was included in the 117 putative target genes listed in the *miR-206* LAMP assay, suggesting that neither gene was a direct target of *miR-206*. Then, using bioinformatics analysis, no corresponding sequences specific for *miR-206* binding were located at the 3′UTRs of *cxcr4a* and *thbs3a*. Based on this line of evidence, it can be concluded that the expressions of *cxcr4a* and *thbs3a* in somites are not directly affected by *miR-206*. Instead, they are regulated by Rtn4a, which is mediated by *miR-206*, as determined in our results.

### 4.5. Rtn4a overexpression results in the loss of somite boundary formation

There are three types of Rtn4a, including Rtn4al, Rtn4am and Rtn4an, which all share 188 amino acid residues at the C-terminal region. However, at the N-terminus, they contain 133, 23 and 7 amino acids, respectively [37]. Interestingly, we found that actin filaments were elongated across the somite boundary if all three Rtn4a subtypes were overexpressed in zebrafish embryos (figure 2), indicating that the functional domain modulating the somite boundary is located at the C-terminus. It is noteworthy that overexpression of Nogo-B, the homologous gene of *rtn4a*, and its Nogo-B receptor can turn on EMT in HeLa cervical cancer cells and breast tumour cells [53,54]. This evidence also supports our findings that overexpression of Rtn4a favours somite cells undergoing EMT, which is unfavourable for epithelialization of boundary cells and, hence, normal somite boundary formation.

### 4.6. Cxcr4a expression affects somite boundary formation

Leal *et al.* [28] reported that inhibition of either SDF-1a or its ligand, Cxcr4, in *Xenopus laevis* embryos resulted in failed somite boundary formation. Since somite separation was not completely formed, myotome elongation and alignment were observed. Nakaya *et al*. [55] also reported that chick Cdc42, one of the Rho family members, is critical for MET processes of somite boundary cells. Unlike the active form of Cdc42 in mesenchymal cells, Cdc42 activity in boundary cells undergoing epithelialization is repressed, suggesting that Rho family activity is also involved in controlling the MET in boundary cells.

When Cxcr4a was inhibited in our zebrafish study, phenotypes such as defective somite boundary and muscle fibre crossover were observed. These phenotypes were similar to those of *Xenopus* embryos injected with *cxcr4*-MO [28]. Furthermore, we demonstrated that inhibition of Cxcr4a resulted in the increase of Thbs3a in zebrafish embryos. Interestingly, when mouse Thbs3a was overexpressed in C2C12 cells, we here also showed that intracellular Cdc42 was present in an active state (electronic supplementary material, figure S7*b*), which was unfavourable for MET. These lines of evidence suggest that boundary cells cannot undergo MET with absent downregulation of Thbs3a and inactivation of Cdc42.

### 4.7. Thbs3a expression affects somite boundary formation

Thbs3a, a secreted ECM protein, belongs to the thrombospondin family. Thrombospondin proteins mainly bind to receptors such as integrin, located on the cell membrane, resulting in the transduction of extracellular signals toward cells [56]. In *Drosophila*, Thbs (Tsp) has vital roles in integrin-dependent ECM organization at developing muscle/tendon attachment sites [57,58]. In zebrafish, Thbs4b is required for muscle attachment. In Thbs4b-deficient embryos, laminin is discontinuous at somite boundaries, suggesting that zebrafish Thbs4b plays a dual role: binding integrin and organizing the tendon ECM at MTJs to maintain muscle attachment [59].

In vertebrates, five *thbs* genes have been reported, including *thbs3*, *thbs4* and *thbs5* categorized as a subclass presenting as homo- and heteropentamers through a conserved coiled-coil structure [60,61]. Although *in vitro* assay demonstrated that Thbs interacts with other integrin ligands, such as laminin, collagen and fibronectin [62], it is still unclear if Thbs plays instructive or merely permissive roles in ECM organization and cell–ECM interactions.

Since Thbs3 and Thbs4 of zebrafish share a similar protein structure [63], it is plausible that Thbs4, together with Thbs3, might form a heterodimeric structure to be functional. However, no evidence was forthcoming in the present study to suggest that zebrafish Thbs3a is in any way integrated with integrin-dependent ECM, leading to the speculation that zebrafish Thbs3a might be involved in maintaining the stability of integrin-dependent ECM because either overexpression or knockdown of Thbs3a expression level caused defective formation of the somite boundary.

Mouse Thbs1 and zebrafish Thbs3a are conserved in two domains, even though they contain different lengths of amino acid residues at the N-terminus [63]. However, they may have distinct functions in different cells. When mouse Thbs1 was added to vascular smooth muscle cells, the degree of intracellular p-FAK was upregulated [64]. By contrast, when zebrafish Thbs3a was overexpressed in somites, the degree of intracellular p-FAK was downregulated. When boundary cells undergo epithelialization, the level of intracellularly active Cdc42 is reduced [55], whereas overexpression of zebrafish Thbs3a results in an increased level of active Cdc42, which is unfavourable for epithelialization. Thus, our study is supported by Lymn *et al*. [64] and Osada-Oka *et al.* [65], who demonstrated that overexpression of Thbs1 favours EMT, resulting in enhanced migration of vascular smooth muscle cells in humans and leading to vascular intimal thickening. In our study, the overexpression of Thbs3a in zebrafish embryos favoured EMT, but normal boundary formation in newly forming somites was halted because it was also unfavourable for epithelialization of boundary cells.

## Supplementary Material

Rtn4-Supplementary data

## Data accessibility

The datasets supporting this article have been uploaded as part of the electronic supplementary material.
