# Supplementary material for: microRNA-206 modulates an Rtn4a/Cxcr4a/Thbs3a axis in newly forming somites to maintain and stabilize the somite boundary formation of zebrafish embryos
Source: Open Biol. 2017 Jul 12;7(7):170009. doi: 10.1098/rsob.170009 (PMC5541343; doi:10.1098/rsob.170009)
Supplement: Rtn4-Supplementary data [file rsob170009supp1.pdf]

electronic supplementary material of "*microRNA-206* modulates an Rtn4a/Cxcr4a/Thbs3a axis in newly forming somite to maintain and stabilize the somite boundary formation of zebrafish embryos "

Microarray analysis	Expression pattern	Genes
Defined genes (85)	Brain (7)	*dixdc1a, LOC100330238, stxbp5a, tmie, wu:fc75a03, zbtb12.2, zgc:162858*
	Heart (5)	*nppa, LOC569706, LOC100330645, epn1, epb41l5*
	Kidney (9)	*csk, fam46c, mapk14b, parn, plcd1b, ppm1a, scamp2l, smg7, zgc:136639,*
	Muscle (23)	*aplp2, cited3, cldna, farp2, gadd45ab, gata4, gtdc1, ldlrad3, m6pr, mef2d, nsd1a, ntn1a, ptprub, rcor1, rrp1, rtn4a, smc1al, srp54, tars, zgc:103508, zgc:154070, zgc:86607, znf142*
	Reproductive system (8)	*LOC559111, mcm9, or105-1, sdhdb, shcbp1, stx8, zgc:112083, zgc:153351,*
	Others (33)	*arhgap20b, clc4, fezf2, glmnb, her3, hypothetical protein LOC402804, hypothetical LOC569837, hypothetical protein LOC797774, hypothetical protein LOC100003579, hypothetical protein LOC100004348, hypothetical protein LOC100148013, il17d, kif17, LOC560607, LOC566814, LOC795145, LOC798327 , nes, rhogb, si:ch73-131e21.5, si:ch211-14c7.2, si:ch211-39h10.1, si:ch211-107m8.2, si:ch211-147a11.7, si:dkey-81n9.1, si:dkeyp-35b8.5, si:zfos-1762d12.1, similar to plcg2, similar to polyductin-L, wu:fk86g06, wu:fb74e01, zgc:91968, zgc:158335*
Undefined genes (32)	—	—

table S1. Using LAMP assay and microarray analysis to search for the putative target genes of *miR-206*. LAMP assay was performed for the cell extracts from zebrafish embryos at 16 hpf, and putative RNAs targeted by *miR-206* were subsequently analyzed by Zebrafish Whole Genome Microarray (Agilent). After analysis of gene expression profile, 117 possible *miR-206* target genes were predicted. Among them, 85 were known genes. Based on the information provided by NCBI and ZFIN databases, we categorized these 85 genes into several groups if they were expressed in the same tissue, such as brain, heart, kidney, muscle, reproductive system, and those that were nonspecific. In this study, we selected four muscle-specific targets, including *cited3*, *gadd45ab*, *rtn4a* and *znf142* (marked in red), for further experiments.

table S2. Loss of function, gain of function and rescue experiments were performed in zebrafish embryos.

Injected concentration	The percentage of embryos with defective somite boundary
Wild type (non treatment)	0% (0/42)
miR-206-MO (8 ng)	56% (19/34)
miR-206-5-mis-MO (8 ng) (control-MO)	0% (0/30)
miR-206-MO (8 ng) + ds *miR-206* RNA (1.5 ng)	29% (12/41)
miR-206-MO (8 ng) + *rtn4a*-MO (2 ng)	9% (3/33)
rtn4al mRNA (400 pg)	94% (31/33)
rtn4al mRNA (200 pg)	67% (16/24)
rtn4al mRNA (100 pg)	20% (6/30)
rtn4al mRNA (200 pg) + *rtn4a*-MO (2 ng)	8% (2/25)
rtn4am mRNA (400 pg)	89% (24/27)
rtn4am mRNA (200 pg)	52% (15/29)
rtn4am mRNA (100 pg)	25% (7/28)
rtn4am mRNA (200 pg) + *rtn4a*-MO (2 ng)	6 % (2/30)
rtn4an mRNA (400 pg)	96% (27/28)
rtn4an mRNA (200 pg)	56% (14/25)
rtn4an mRNA (100 pg)	25% (6/24)
rtn4an mRNA (200 pg) + *rtn4a*-MO (2 ng)	4% (1/27)
cxcr4a-MO (4 ng)	70% (21/30)
thbs3a mRNA (800 pg)	90% (28/31)
thbs3a mRNA (400 pg)	61% (22/36)
thbs3a mRNA (200 pg)	42% (13/31)
thbs3a mRNA (400 pg) + *thbs3a*-MO (1 ng)	22% (6/27)

The percentage of embryos with defective somite boundaries among the examined embryos were calculated at 48 hpf.

(i) Injection of MO (morpholino; antisence oligonucleotide) served as loss of function experiment.
(ii) Injection of mRNA (*rtn4al, rtn4am, rtn4an* and *thbs3a*) served as gain of function experiment.
(iii) Injection of mRNA combined with MO (*miR-206*-MO + ds *miR-206* RNA, *rtn4al* mRNA + *rtn4a*-MO, *rtn4am* mRNA + *rtn4a*-MO, *rtn4an* mRNA + *rtn4a*-MO and *thbs3a* mRNA + *thbs3a*-MO) and injection of *miR-206*-MO combined with *rtn4a*-MO served as rescue of function experiment.

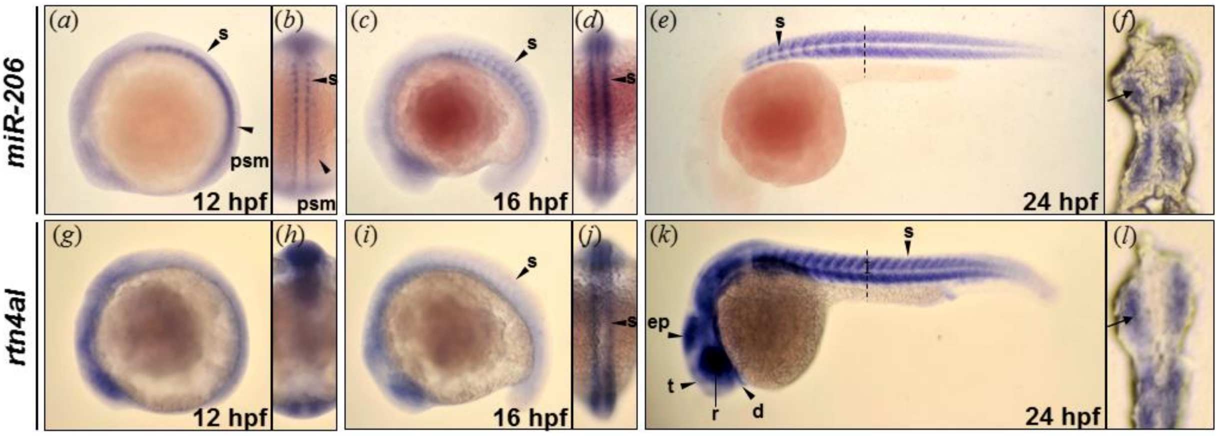

figure S1. Expression patterns of *miR-206* and *rtn4al* in zebrafish embryos at different developmental stages. Using WISH to detect the temporospatial expression patterns of (*a-e*) *mir-206* and (*g-l*) *rtn4al* in zebrafish embryos at 12, 16 and 24 hpf, as indicated. F and L are cross section, (*a*,*c*,*e*,*g*,*i*,*k*), lateral views; (*b*,*d*,*h*,*j*), dorsal views; and (*f*,*l*), transversal views. (*a-f*) *miR-206* expression at 12, 16, and 24 hpf. *miR-206* was specifically expressed in presomitic mesoderm (psm), somite (s) and in fast fibrils of muscle in trunk (arrow). (G-L) *rtn4al* was initially detected in somite at 16 hpf. Additionally, *rtn4al* was expressed in retina (r), epiphysis (ep), telencephalon (t), diencephalon (d) and fast fibrils of muscles in trunk at 24 hpf (arrow).

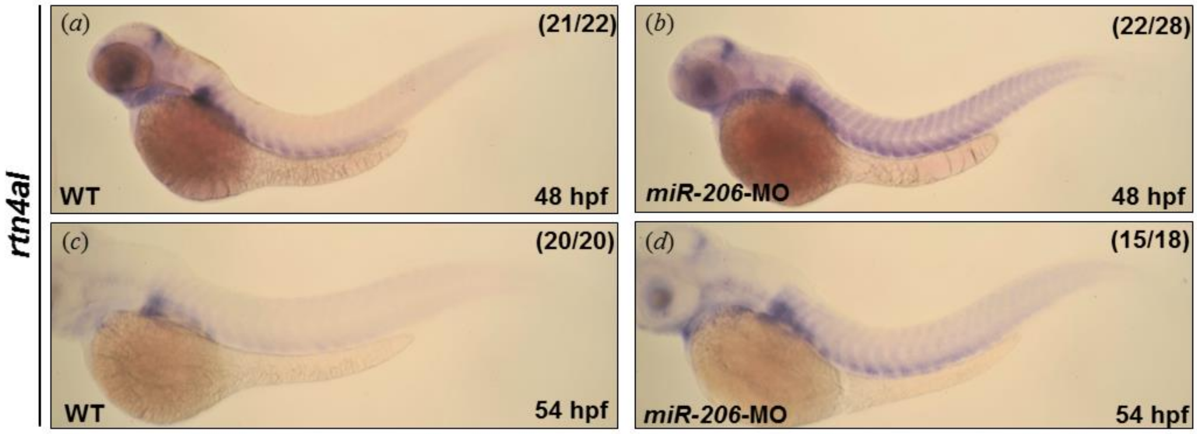

figure S2. Knockdown of endogenous *miR-206* increased the amount of *rtn4al* mRNA in zebrafish embryos. (*a*,*c*) Wild type (WT) embryos; (*b*,*d*) knockdown of endogenous *miR-206* by injection of *miR-206*-MO in embryos. The expression level of *rtn4al* mRNA was detected at 48 and 54 hpf, as indicated.

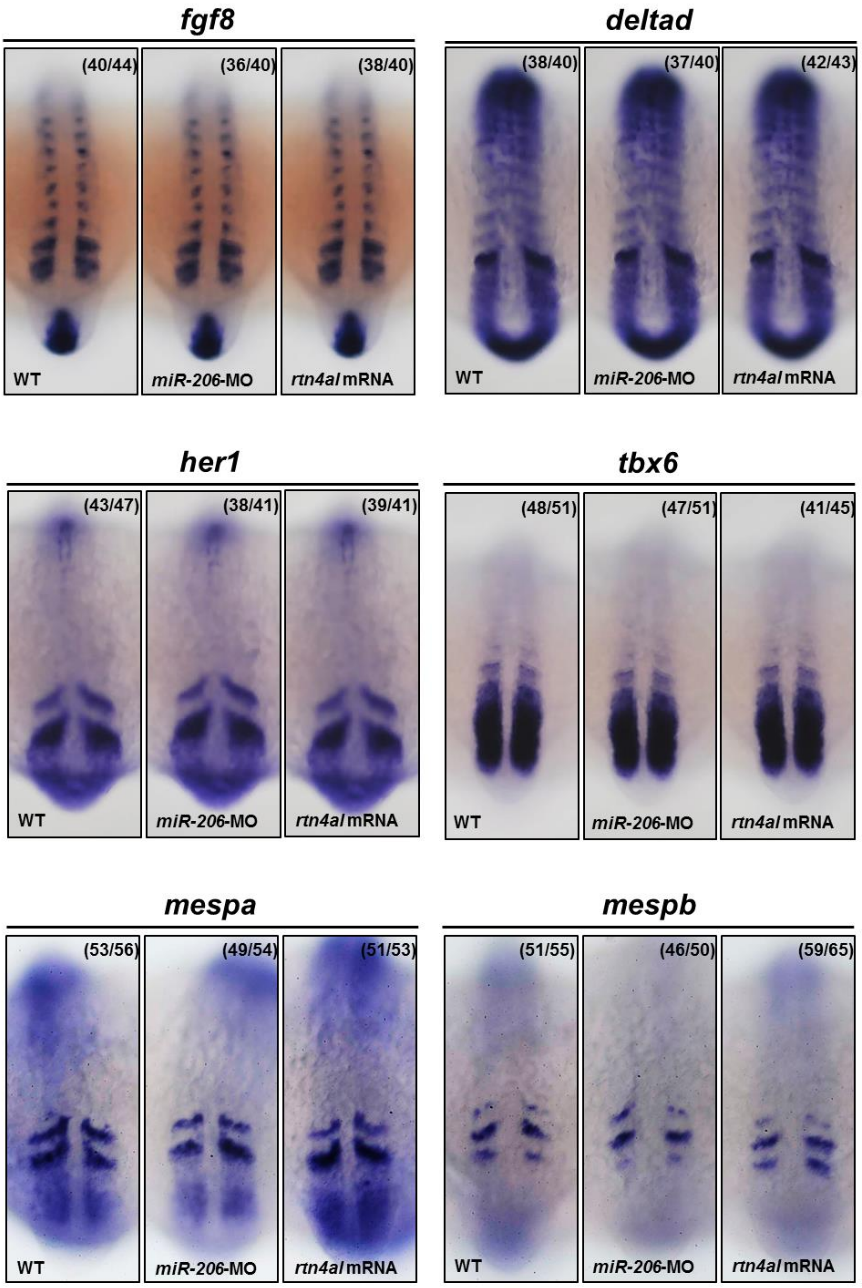

figure S3. Neither knockdown of *miR-206* nor overexpression of *rtn4al* mRNA affected the expression levels of regulatory genes involved in somitogenesis in zebrafish embryos. Wild-type (WT) embryos, *miR-206*-MO-injected embryos and *rtn4al*-mRNA-injected embryos were developed at 16 hpf. Using WISH to detect the transcript levels of *fgf8, deltad, her1, tbx6, mespa,* and *mespb* in zebrafish embryos at 16 hpf. The numbers shown at the upper-right corner represent the number of phenotypes out of the examined embryos.

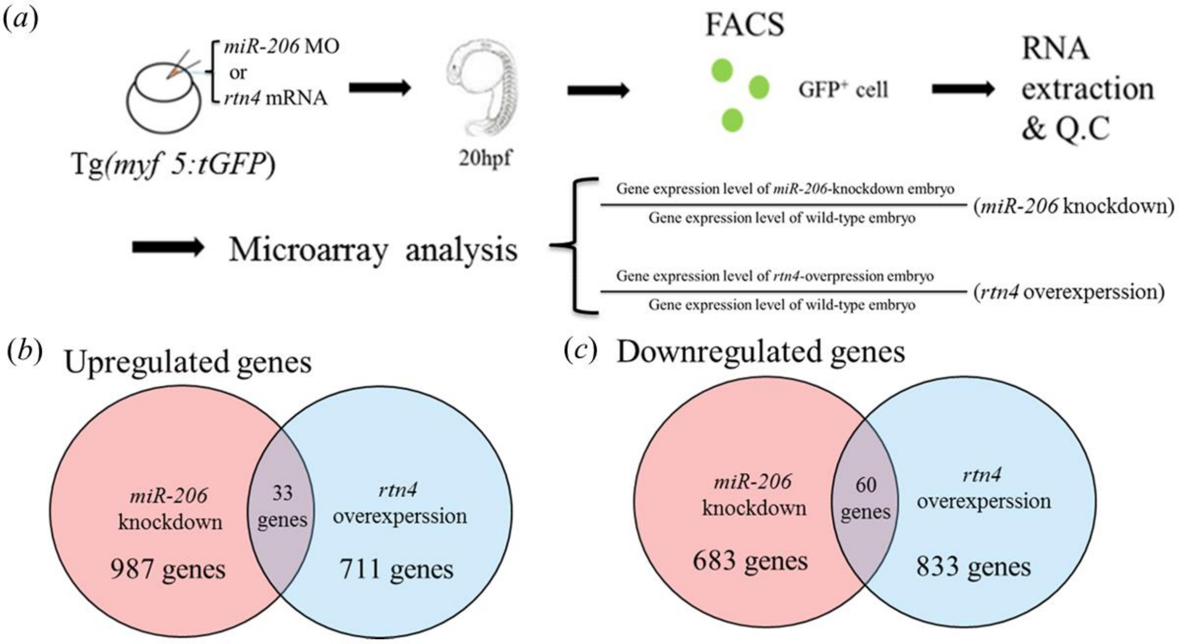

figure S4. Microarray analysis of putative genes having corresponding changes in zebrafish embryos treated with *miR-206*-knockdown and *rtn4al*-overexpression. (*a*) Injection of *miR-206*-MO or *rtn4al*-mRNA in the one-cell embryos derived from Tg(*myf5*:GFP), in which GFP is driven by zebrafish *myf5* promoter and specifically expressed in somites. At 20 hpf, fluorescence- activated cell sorting (FACS) was employed to collect GFP-expressing cells [GFP(+)], followed by extraction of total RNAs to perform microarray analysis. (*b*) Expression of 987 genes was increased in *miR-206*-knockdown embryos, while expression of 711 genes was increased in Rtn4al-overexpression embryos. In total, 33 genes showed increased expressions in both *miR-206*-knockdown and *rtn4al*- overexpression embryos. (*c*) Expression of 683 genes was decreased in *miR-206*-knockdown embryos, while expression of 833 genes was decreased in Rtn4al-overexpression embryos. In total, the expression of 60 genes was decreased in both *miR-206*-knockdown and *rtn4al*- overexpression embryos.

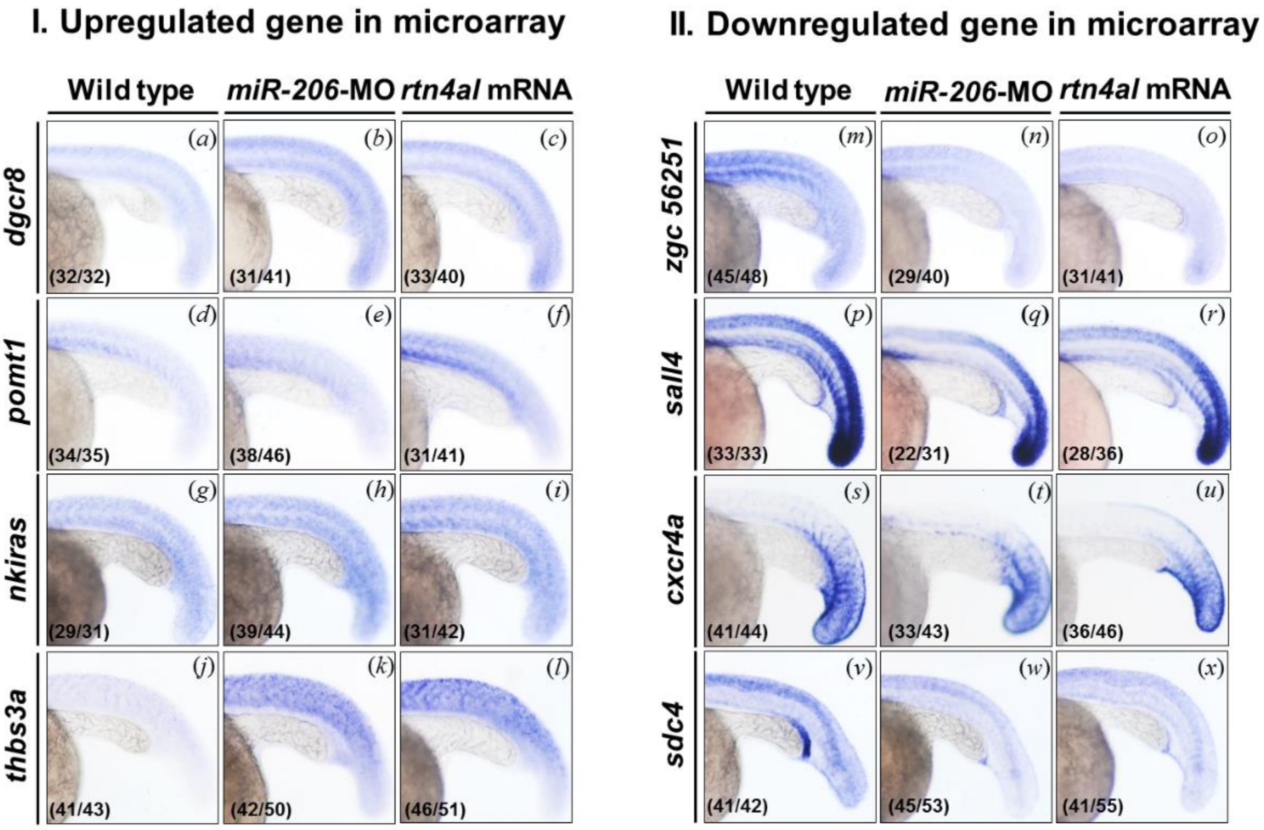

figure S5. Expression patterns of some putative genes obtained from microarray analysis of *miR-206*-knockdown and *rtn4al*-overexpression zebrafish embryos. Using WISH to detect the gene expression patterns of four upregulated genes and four downregulated genes in WT embryos (*a,d,g,j,m,p,s,v*), *miR-206*-MO-injected embryos (*b,e,h,k,n,q,t,w*), and *rtn4al*-mRNA-injected embryos (*c,f,i,l,q,r,u,x*) at 20 hpf. These examined genes were specifically expressed in the somites of zebrafish embryos. Compared to WT embryos, the expression levels of (*a-c*) *dgcr8*, (*d-e*) *pomt1*, (*g-i*) *nkiras2,* and (*j-l*) *thbs3a* were increased in the *miR-206*-MO-injected and *rtn4al*-mRNA-injected embryos, while those of (*m-o*) *zgc56251*, (*p-r*) *sall4,* (*s-u*) *cxcr4a,* and (*v-x*) *sdc4* were decreased. Numbers shown at the lower-left corner were the numbers of phenotypes out of the examined embryos.

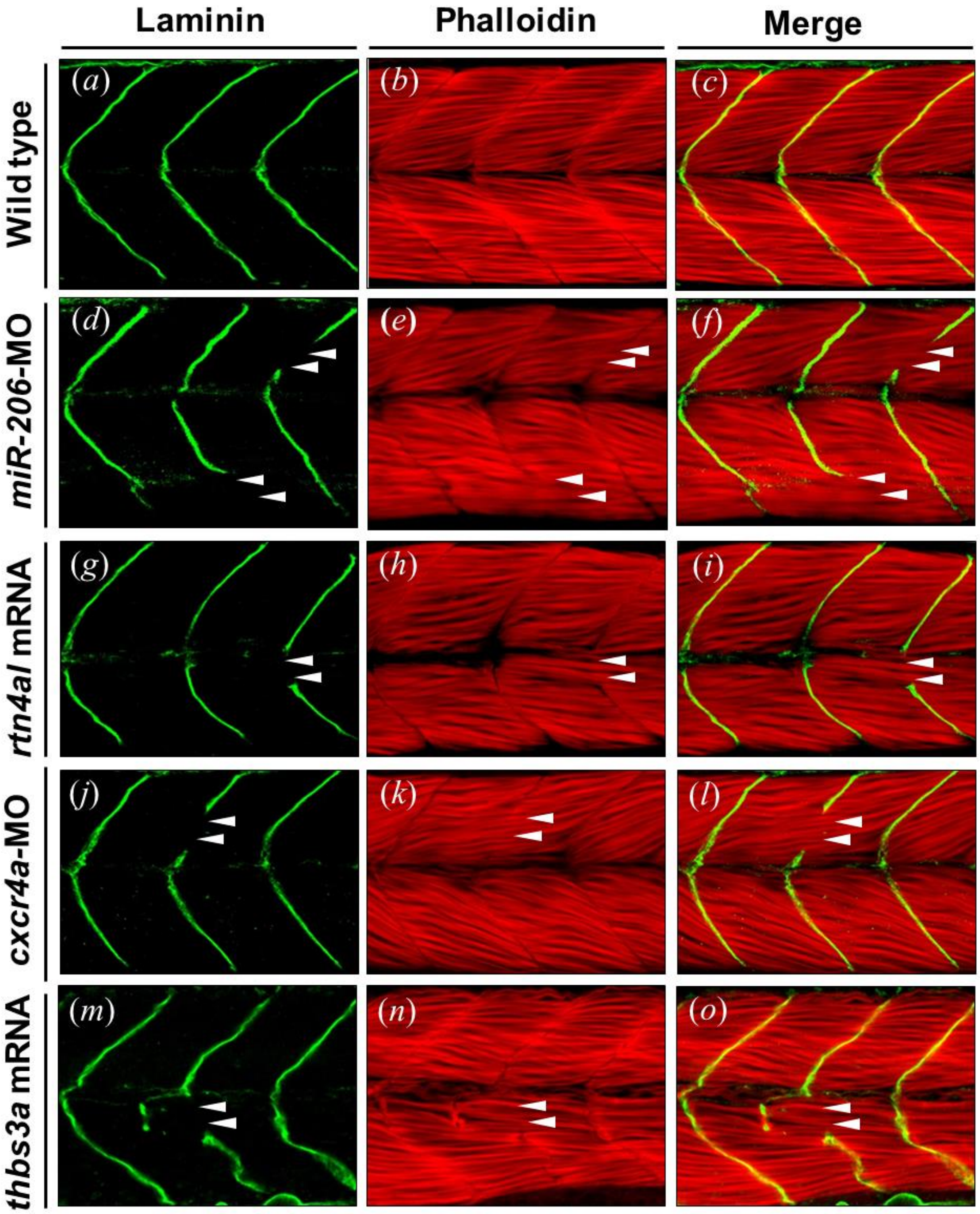

figure S6. Defective somite boundary caused by knockdown of either *miR-206* or *cxcr4a* or overexpression of either *rtn4al* or *thbs3a* was not restored at late developmental stage of zebrafish embryos. Immunostaining was performed for (*a*-*c*) WT embryos, (*d*-*f*) *miR-206*-MO-injected embryos, (*g*-*i*) *rtn4al*-mRNA-injected embryos, (*j*-*l*) *cxcr4a*-MO-injected embryos and (*m*-*o*) *thbs3a*-mRNA-injected embryos at 48 hpf. (*a*,*d*,*g*,*j*,*m*) Green fluorescence-labeled Laminin was used to detect

somite boundary; (*b*,*e*,*h*,*k*,*n*) Red fluorescence-labeled Phalloidin was used to detect F-actin; and (*c*,*f*,*i*,*l*,*o*) merged images of these two fluorescent signals. Some muscle fibers grew transversely across the somite boundary where the boundary was defective, as indicated by white arrowheads.

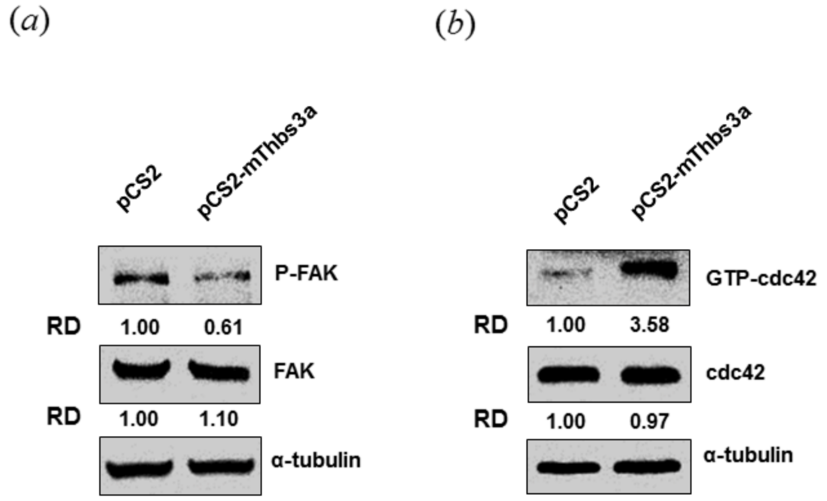

figure S7. Overexpression of mThbs3a in the undifferentiated C2C12 cells resulted in the decrease of p-FAK level and the increase of active cdc42 level. (*a*) Overexpression of mThbs3a caused the reduction of intracellular p-FAK[pY397] level in C2C12, but (*b*) the increase of intracellular active cdc42 level through pull-down detection. RD: Relative density.
